# A novel computational strategy to predict the value of the evidence in the SNP-based forensic mixtures

**DOI:** 10.1371/journal.pone.0247344

**Published:** 2021-10-15

**Authors:** Vincenzo L. Pascali

**Affiliations:** Department of Healthcare Surveillance and Bioethics, Catholic University, Rome, Italy; University of Politehnica of Bucharest, ROMANIA

## Abstract

This study introduces a methodology for inferring the weight of the evidence (WoE) in the single nucleotide polymorphism (SNP)-typed DNA mixtures of forensic interest. First, we redefined some algebraic formulae to approach the semi-continuous calculation of likelihoods and likelihood ratios (LRs). To address the allelic dropouts, a peak height ratio index (“h,” an index of heterozygous state plausibility) was incorporated into semi-continuous formulae to act as a proxy for the “split-drop” model of calculation. Second, the original ratio at which a person of interest (POI) has entered into the mixture was inferred by evaluating the DNA amounts conferred by unique genotypes to any possible permutation of any locus of the typing protocol (unique genotypes are genotypes that appear just once in the relevant permutation). We compared this expected ratio (MR_ex_) to all the mixing ratios emerging at all other permutations of the mixture (MR_obs_) using several (1 - χ^2^) tests to evaluate the probability of each permutation to exist in the mixture according to quantitative criteria. At the level of each permutation state, we multiplied the (1 - χ^2^) value to the genotype frequencies and the h index. All the products of all the permutation states were finally summed to give a likelihood value that accounts for three independent properties of the mixtures. Owing to the (1 - χ^2^) index and the h index, this approach qualifies as a fully continuous methodology of LR calculation. We compared the MRs and LRs emerging from our methodology to those generated by the EuroForMix software ver. 3.0.3. When the true contributors were tested as POIs, our procedure generated highly discriminant LRs that, unlike EuroForMix, never overcame the corresponding single-source LRs. When false contributors were tested as POIs, we obtained a much lower LR value than that from EuroForMix. These two findings indicate that our computational method is more reliable and realistic than EuroForMix.

## 1. Introduction

In the last two decades, multi-allelic polymorphisms (essentially short tandem repeats; STRs) have been widely used in the routine casework of forensic genetics. Single nucleotide polymorphisms (SNPs) have recently emerged as promising polymorphism to be adopted in this field. SNPs are mostly biallelic, and the Mendelian distribution of their genotypes is determined by sampling only a handful of unrelated individuals. SNPs are practically ubiquitous; they can be typed *en masse* and their current typing procedures (next-generation sequencing; NGS) are unaffected by technical artifacts that normally complicate the process of STR typing [1–2]. Finally, several SNP panels have been assembled for human identification and validated for forensic routine [3–20].

However, the routine application of SNP panels to the field of criminal identification has proceeded at a slow pace because of a series of circumstances:

- The calculation procedures used to weigh the value of the evidence (WoE) in forensic genetics (qualitative/semi-continuous likelihood ratios (LRs) [21]; quantitative LRs [22–27]) have been developed for protocols targeting a few STR loci with multiple alleles, and they need to be readapted to the case of hundreds or thousands of SNPs, each with two alleles.
- Many forensic stains are mixtures, and even when the number of contributors is high, SNPs give back invariably monoallelic/biallelic evidence. Based on this evidence, one cannot formally exclude any specific suspect, and this raises the serious problem of discriminating falsely compatible results from genuine matches by a statistical approach and not by the test’s formal outcome.

Consequently, in most countries, SNPs are seldom used to set up DNA identity tests, with only sporadic exceptions [28]. The “computational” bottleneck at SNP mixtures analysis has recently attracted the attention of several scholars who have introduced methods diverging from the classical LR approach. For example, estimates based on the absolute likelihood have been proposed as a computational complement to mass typing protocols [29, 30]. This “frequentist” approach has been further developed [31] and criticized [32, 33]. Furthermore, other authors [34] used a mass genomic typing protocol based on 3,000 low-polymorphic content SNPs (with 0.002 < P (MAF) < 0.250; MAF is the allele with minor gene frequency) and succeeded in identifying a single contributor by a very high WoE.

Nearly at the same time, the feasibility of the classical semi-continuous or qualitative LR calculations in the case of 2-person and 3-person mixtures was explored [35]. In turn, more recently, the open-source EuroForMix software has been readapted to the biallelic polymorphisms so as to perform the quantitative LR calculations starting from the”reads” produced by the massive parallel sequencing (MPS) equipment [36].

Herein, we will revisit the logic of calculating the biallelic likelihoods and LRs in forensic mixtures, from both the semi-continuous and quantitative perspectives. We will introduce general formulae for *semi-continuous* likelihoods and a novel approach for the calculation of *continuous* LRs, based on population genetics frequencies and on Mendelian and quantitative probability indexes. Our purpose is innovating and improving all computational procedures currently used to extract the WoE in the SNPs mixtures.

## 2. Materials and methods

### 2.1. Genotypic matrices and likelihoods

Mixtures are a class of synthetic forensic evidence (E) contained in a biological trace and supposed to originate from a collection of “n” individual genotypes (n > 1; n is also the number of individuals involved in a crime and leaving their DNA in the trace). Mixtures based on the autosomal STRs have multiple patterns of evidence—each pattern is denoted by a definite number of alleles—and a variable number of contributors, each of whom confers the trace one or two alleles (depending on their homozygous or heterozygous condition). When nothing else is a priori known about the trace content except the number of contributors, the evidence should be explained by a series of equally possible combinations of genotypes elicited from classical combinatorial work. If, for some reason, the order in which these genotypes are listed in each combination matters (as in the case of quantitative likelihood calculations), combinations turn into permutations, and the number of admissible records equally accounting for the same evidence increases. Therefore, mixtures may be conveniently represented as genotype permutation matrices, comprising as many columns as the alleged contributors and as many records as the permutation states embodying the evidence.

Mastering the combinatorial work of STR mixtures involves harnessing a system with multiple matrices and thousands or tens of thousands states, a task that may be challenging and error-prone [37]. By contrast, the combinatorial work involving SNP mixtures is simple. Here, the evidence may only be a single-allele E{a} or double allele E{a;b}. With E{a}, no matrix is generated. Nonetheless, E{a;b} generates only one matrix for each number of contributors, all with an acceptable number of permutations (one can count 7 permutation records at the 2PM matrix, 25 records at the 3PM matrix, 79 at the 4PM, 249 at 5PM, 727 at 6PM, 2185 at 7PM, and 6559 at 8PM). If a minimal, standard nomenclature is adopted for the combinatorial items, only one matrix will be suitable for whatever locus of interest. Here, for example, we will adopt: “a” for the quantitatively predominant allele, “Q_a_”for the number of its DNA molecules, “f_a_” for its gene frequency; “b,” for the minor quantity allele, “Q_b_” for its DNA quantity, “f_b_” for its gene frequency; AA, AB, and BB for the biallelic genotypes; Q_AA_, Q_AB_, Q_BB_ for their quantity; P(AA) = f_a_^2^, P(AB) = 2f_a_f_b_, P(BB) = f_b_^2^ for their probability in the reference population. In our matrices, the alleles at all loci of the protocol were redenominated in the same way by changing the original wildtype major/mutant minor allele frequency (MAF) nomenclature most often found in the literature: as expected, the genotypes behaved accordingly (e.g., Q_AA_ > Q_BB_).

Permutation matrices generated by E{a;b} under the only prior hypothesis of knowing “n” and no more than n represent the most generic way of interpreting mixtures and the most basic likelihood to calculate. All individuals contributing to the trace are here regarded as unknown (here we will use a ‘U‘ to denominate every single unknown individual within a synthetic likelihood formula).

When further prior hypotheses are formulated introducing η persons of interest (POIs; also denominated as “known contributors”; with 1 ≤ η ≤ n; we will use a letter ‘K’ to denominate each individual POI placed within a synthetic likelihood formula), the inference to draw becomes conditional on both n and η [38]. As noted for E, the POI reference sets can have a single or biallelic pattern (K{a}; K{a;b}). A POI genotype is used to transform the all unknown, general matrix (a likelihood with n Us) into one with few permutations (only those permutations that contain the POI genotype; synthetically: a likelihood with one K and (n-1) Us). More than one POI with further restrict the number of selected permutations. For practical examples of how to reproduce this procedure on a worksheet, the reader should refer to some matrices (NITZq_SNPs_2PM_upl.xlsx; NITZq_SNPs_3PM_upl.xlsx; NITZq_SNPs_4PM_upl.xlsx) to be found in the S1 File of this article.

### 2.2. Semi-continuous likelihoods

**Fig 1:**
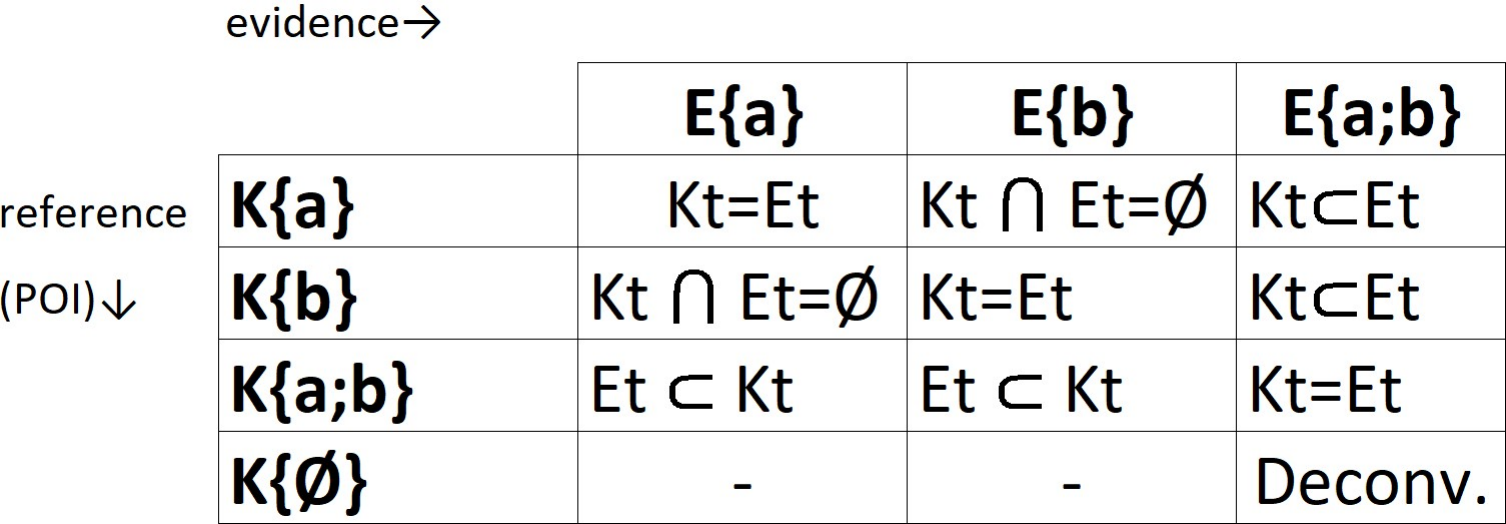
Assortments of the evidence and the POI (person of interest) allelic subsets. A limited number of outcomes (just nine, in fact) are expected at a SNP-based identity test. (‘Deconv.’ refers to a permutation matrix with no POI available).

When compared to one another, the evidence and the POI reference sets generated nine assortments, as shown in Fig 1; this figure also shows the matching cases and exclusions. Exclusions only emerged when the evidence had a single allele. When this happens, either K and E are disjointed sets (K ⋂ E = Ø; no allele in common; for example, E{a} comparing to K {b} and vice versa) or E is a subset of K: E ⊂ K (whereas in the case of compatibility one would normally expect the reverse K ⊂ E to be true).

All of these nine cases may be solved according to the binomial properties:

1. at a single-source trace, the whole evidence is: PE{a;b} = (f_a_+f_b_)^2^ = f_a_^2^+ f_b_^2^ +2f_a_f_b_ = 1;
2. the generalization of the single-source trace model to n contributors requires exponentiation to n of all members of the equation: PE{a;b} |n) = f_a_^2n^+ f_b_^2n^ + (2f_a_f_b_)^n^ [21];
3. introducing η POIs and calculating the relevant likelihoods involves subtracting η to the exponent: PE{a;b} |n, η) = f_a_^2(n-η)^+ f_b_^2(n-η)^+ (2f_a_f_b_) ^(n-η)^.

#### 2.2.1. Compatible semi-continuous likelihoods

The following conditional formulas apply to compatible cases of Fig 1:

$$P(E\{a\}|K\{a\},n,\eta )=f_{a}{}^{2(n‐\eta )}$$ (1)

$$P(E\{b\}|K\{b\},n,\eta )=f_{b}{}^{2(n‐\eta )}$$ (2)

$$P(E\{a,b\}|K\{a\},n,\eta )=1‐f_{a}{}^{2(n‐\eta )}$$ (3)

$$P(E\{a;b\}|K\{b\},n,\eta )=1‐f_{b}{}^{2(n‐\eta )}$$ (4)

P(E|E{a;b},K{a,b},n,η)=1 (5)

$$P(E|K\{a;b\},n)=1‐fa^{2n}‐f_{b}{}^{2n}$$ (6)

These formulae are a generalization of those previously reported [34] and they have been modified by the introduction of rule 3 described in the previous section. As previously noted by these authors, these six formulae do not account for cases where there is a mismatch between the evidence and the POI.

#### 2.2.2. Incompatible likelihoods

We need to assume that the monoallelic evidence derives from originally biallelic evidence by the loss of one allele (allele dropout). Within our nomenclature system, only the minor quantity allele can drop out of evidence, with some probability (P(D)) that is inversely proportional to the template concentration. In the available literature, dropouts are “stochastic events”, and the P(D) values have been inferred from experiments conducted on various template dilutions using logistic regression analysis [39, 40]. The resulting experimental values were incorporated into the semi-continuous calculations. A widely used computational approach relying on such dropout probabilities is the “split-drop approach” [41]. The split drop approach was readapted to the case of SNPs [34] and is now adopted by the two widespread software LRMIX [35] and EuroForMix [36].

Instead of using the split drop approach, we will incorporate dropout probabilities (denoted by the letter ‘h’) into some of our algebraic formulae:

$$P(E\{a_{d};b\}|K\{a\};P(h);n;\eta )=h^{2}{}_{*}f_{a}{}^{2(n‐\eta )}$$ (7)

$$P(E\{a;b_{d}\}|K\{b\};P(h);n;\eta )=h^{2}{}_{*}f_{b}{}^{2(n‐\eta )}$$ (8)

$$P(E\{a;b_{d}\}|K\{a;b\};P(h);n;\eta )=h\;or:$$

$$P(E\{a_{d};b\}|K\{a;b\};P(h);n;\eta )=h$$ (9)

It should be noted that, with reference to Fig 1, E{a;b_d_} replaces the original E{a} notation and E{a_d_;b} replaces E{b}. P(h) is our proxy for the probability of the minor allele b dropping out of the evidence, replacing the experimental logistic regression values. For its meaning, importance and use the readers are referred to the following paragraphs.

#### 2.2.3. Dropped out and still compatible cases

Although the concept of dropout is used to denote cases of contributors that do not match the evidence, “exclusion” and “dropout” are not logically equivalent. There may be instances where an allele drops out of the evidence, but the POI remains compatible. Such “latent” dropout cases can be modeled by turning Eqs 1 and 2 into:

$$P(E\{a;b_{d}\}|K\{a\},n,\eta )=\left(1‐h\right)*f_{a}{}^{2(n‐\eta )}$$ (1D)

$$P(E\{a_{d};b\}|K\{b\},n,\eta )=\left(1‐h\right)*f_{b}{}^{2(n‐\eta )}$$ (2D)

It should be noted that, as long as our conventional nomenclature (a, major read allele; b, minor-read allele) holds, Eq 2D is never actually used (“a” cannot drop out unless “b”‘ is first dropped; if both drop the whole evidence disappears and Eq 2D becomes useless).

#### 2.2.4. Re-defining the inferential table

**Fig 2:**
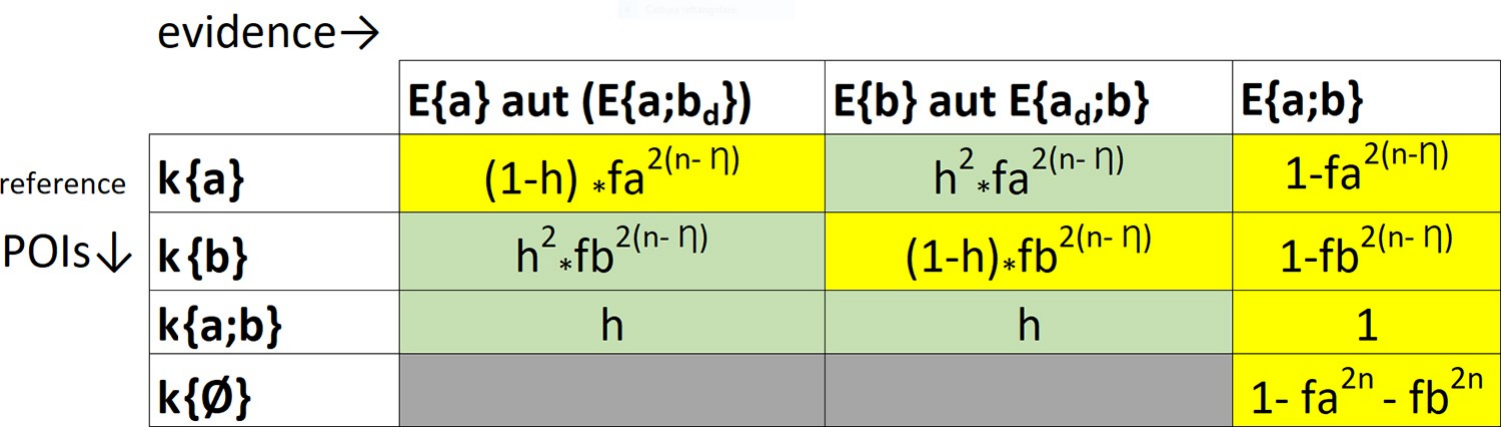
The algebraic formulae for all possible evidence/POI allelic sets assortments of Fig 1. The monoallelic evidence in Fig 1 is re-interpreted under the assumption that dropout events may have altered the original evidence E{a;b}. The compatible cases are highlighted in yellow background and the exclusions are highlighted in green background.

The algebraic solutions we have just introduced (1d, 2d, and 3–9) can now cover all the inferential scenarios expected to occur at any test based on biallelic markers. An overview of the nine relevant cases and formulas is shown in Fig 2.

#### 2.2.5. The h index as a proxy for experimental P(D) values

Dropout also occurs at the SNP level [42], but specific dropout values are not available for all SNPs of forensic interest. This is not surprising as SNPs are very numerous. Their P(D) values should be inferred (by treating experimental data with logistic regression) from each locus in use at a given protocol, possibly by each laboratory adopting the protocol, and the relevant task is not easy to achieve. For this reason, a good deal of semi-continuous calculations at SNP mixtures (as those performed by typical users of LRMIX and EuroForMix) tend to rely on P(D) values that are arbitrary. This encourages the search for other solutions.

We found that a convenient proxy for P(D) is the h index [43], in its simplest form (the minor read simply divided by the major read; at the NGS technology, a read is the equivalent of a peak height in an STR electrophoretic diagram). There are several reasons to introduce this index instead of logistic regression-derived P(D) values:

- h applies to single-source stains and mixtures as well.
- In mixtures, h may represent the entire propensity of the minor-read allele to drop out of the mixed evidence.
- (1-h) can estimate the probability of the existence of two-allele evidence.
- It has been noticed [44] that a P(D) can automatically emerge from the peak height analysis, without any external logistic regression modeling; P(D) values emerging from the peak area analysis can only be the h index.

The index h enters the likelihood formulae, as shown in the previous paragraphs. We will further use it for quantitative calculations as well.

#### 2.2.6. Dropins

According to the definition issued by a commission of the International Society for Forensic Genetics [45], a dropin is an allele that is not associated with the crime sample and remains unaccounted for both under the prosecutor and defense hypothesis. In principle, a dropin phenomenon is nothing more than an additional contributor to a mixture, and its case can be modeled by increasing the number of contributors. Because the SNP-based evidence generates relatively simple permutation matrices even with high numbers of contributors, we will address the dropin case by systematically switching the current scheme of computation to one scheme with one more contributor.

#### 2.2.7. Number of contributors

Predicting the number of contributors entering a SNP mixture may be difficult. The importance of the whole issue is directly proportional to the impact that the value of n may have over the LR values. This issue was explored by simulating 100 mixtures with two to up to six virtual contributors and calculated likelihoods at various quantitative POI proportions [36]. It was consequently found that when the POI is not quantitatively predominant, their two-person mixture model of analysis would return them higher LRs than those with the three- or more-person models. They also found that, from three-person mixtures onwards, increasing the number of contributors has a negligible effect on LR of POIs, regardless of its quantitative predominance. We have an explanation for this phenomenon: unlike higher-rank mixtures, 2-person mixtures cannot have permutation states with the full assortment of biallelic genotypes (AA + AB + BB). Switching from 2PM to 3PM has, therefore, the effect of completing the genotype assortment, elongating each permutation, increasing the number of permutations, increasing the uncertainty and lowering the LR. The same does not occur when switching from 3PM to 4PM or higher-rank models. Consequently, it is logical to believe that 3PM is the minimum standard for biallelic mixture analysis. In this study, however, we would always calculate LRs under three distinct hypotheses (2PM, 3PM, and 4PM).

#### 2.2.8. LRs

Each likelihood in Fig 2 may represent the viewpoint of prosecution or defense in a typical judicial test. A ratio between two of these likelihoods will return the value of the available prevailing evidence. The two parties may choose to opt for the same or different n values, but they usually diverge on the choice of the η value. The prosecutor’s choice η1, placed in the numerator of a ratio, is always higher than the defenses’ choice η2 placed in the denominator ((η1- η2) ≥ 1). In this paper, the classical LR formula [46] is therefore written as:

$$LR=\frac{Pr(E|Hp,n1,\eta 1)}{Pr(E|Hd,n2,\eta 2)}$$ (10)

### 2.3. Quantitative likelihoods

#### 2.3.1. An empirical, quantitative approach

The issue of quantitative mixture analysis has been reviewed several times [47–51] and according to the opinion of most authors, a key aspect of this issue is finding a suitable way to infer the peak fraction values pertaining to each contributor from the whole evidence. In a pioneering study [26], the gamma distribution was used to deduct all the individual peak height fractions that coalesce into the mixed STR profile. Concurrently, the Markov chain Monte Carlo (MCMC) simulations were introduced to infer the same information [23]. These two methods have earned much relevance in the 2010–2020 decade, and presently numerous computer programs calculate LRs based on the gamma model or MCMC simulations.

Herein, we adopt a novel empirical method for calculating likelihoods based on quantitative evidence. The rationale of our method is as follows:

First, regardless of any available POI, we will quantitatively denote each genotype of each permutation of each locus of interest by converting the synthetic locus-specific evidence into individual genotypic quantity [E(Q_a_;Q_b_)→ E(Q_AA_; Q_AB_;Q_BB_)] according to basic Mendelian rules. Starting from these individual DNA amounts, we will assign a mix ratio (MR_obs_) to each observed permutation. With specific reference to the calculation examples we report in this study, a 2PM protocol based on 133 loci will have 931 MR_obs_; a 3PM protocol will have 3,335.

Second, when a POI is available, we will estimate the quantitative contribution of the POI to the mixture by selecting the POI unique genotypes (a unique genotype is a genotype that appears just once in the permutation) to be found at each locus permutation of the protocol, by summing the relevant DNA amounts and dividing this sum by the total DNA amount in the protocol. An overall mix ratio (MR_ex_) is obtained. There will be only one MR_ex_ per mixture protocol, and MR_ex_ will be regarded as the ratio expected to invariably show up at each locus when the POI is the true trace contributor.

Finally, we give each permutation state appearing at each locus a distinctive probability to exist based on a (1 - χ^2^) statistical test. To follow the previously cited examples, 931 tests will be performed at the 2PM/133 loci and 3,335 (1 - χ^2^) tests at 3PM. At each (1 - χ^2^) test, MR_ex_ will be the expected MR dataset, and the permutation-specific MR_obs_ will be the observed datasets. Calculating (1-χ^2^) is an essential step in the process of calculating quantitative likelihood values.

#### 2.3.2. Giving MRs to all permutation states

As a first step in quantitative calculation, the synthetic evidence E{Q_a_;Q_b_} is to be converted into genotypic evidence E {Q_AA_;Q_AB_;Q_BB_} by using rules that can apply to all mixture schemes. We found that, regardless of the contributor number, the most parsimonious genotype combinations generating the E{a;b} evidence are:

rAA+sBB, with n = r+s

rAA+sAB, with n = r+s

rAA+sBB+tAB, with n = r+s+t (not applicable to a 2PM)

rAB+sBB, with n = r+s

nAB

These five combinations account for all possible mixture permutations, whatever the number of contributors may be. Because apportioning individual quantities to identical genotypes is impossible, groups of identical genotypes have to be assigned just one DNA amount. The following rules will consequently enable to calculate the mixture ratio everywhere:

1. rAA+ sBB; Q_rAA_ = [Q_a_/(Q_a_+Q_b_)]; Q_sBB_ = [Q_b_/(Q_a_+Q_b_)]
2. rAA+sAB; Q_rAA_ = [(Q_a_-Q_b_)/(Q_a_+Q_b_)]; Q_sAB_ = [2Q_b_/(Q_a_+Q_b_)];
3. rAA+sAB+tBB; Q_sAB_ = {[Q_a_^2^/(Q_a_+Q_b_)] + [Q_b_^2^/(Q_a_+Q_b_)]}; Q_rAA_ = [(Q_a_+Q_b_)—Q_AB_]; Q_tBB_ = [(Q_a_+Q_b_)—Q_AB_];
4. rAB+sBB; Q_sBB_ = [(Qb_*_2s)/(2s+r)]; Q_rAB_ = [(Q_a_+Q_b_)- Q_BB_]
5. nAB; Q_nAB_ = (Q_a_+ Q_b_)

With the exception of rule 4, these rules are based on elementary autosomal Mendelian principles and they return the same mixture ratio value whatever the number of contributors may be. A network of quantities is consequently distributed to all permutation states, as it is shown in the three matrix examples (NITZq_SNPs_2PM_upl.xlsx; NITZq_SNPs_3PM_upl.xlsx; NITZq_SNPs_4PM_upl.xlsx) to be found in the S1 File of this article.

#### 2.3.3. Finding the ‘expected’ mixture ratio of a trace

In SNP matrices, the genotypes have to overlap at individual alleles (**AA** + **AB; AA** + **AB** + **BB**) and to be redundant (for example AA+AA; AB+AB; BB+BB; “redundant” is here said of a genotype that appears more than once within the same combination) to make room for additional contributors. When contributors grow in number, the relevant combinations generate overlaps and redundancies of various orders of complexity. However, even within the context of heavy genotype stacking, a network of **unique genotypes** persists regardless of the mixture type. Unique genotypes are genotypes that share their DNA amount with no other and are of two types:

1. non redundant-non overlapping genotypes (**NOGs;** with parsimonious structure AA+(n-1)BB; (n-1)AA+BB); here if the unique genotype is BB then Q_BB_ = Q_b_; if the unique genotype is AA, then Q_AA_ = Q_a_.
2. Non redundant-simply overlapping genotypes (**SOGs**; with structure **AA**+ (n-1)**AB; (n-1)AA+AB**). Here if the unique genotype is AB, the ‘B’ half quantity is Q_b_ and Q_AB_ = 2Q_b_.; if AA is unique, then Q_AA_ = Q_a_–Q_b_.

**Fig 3:**
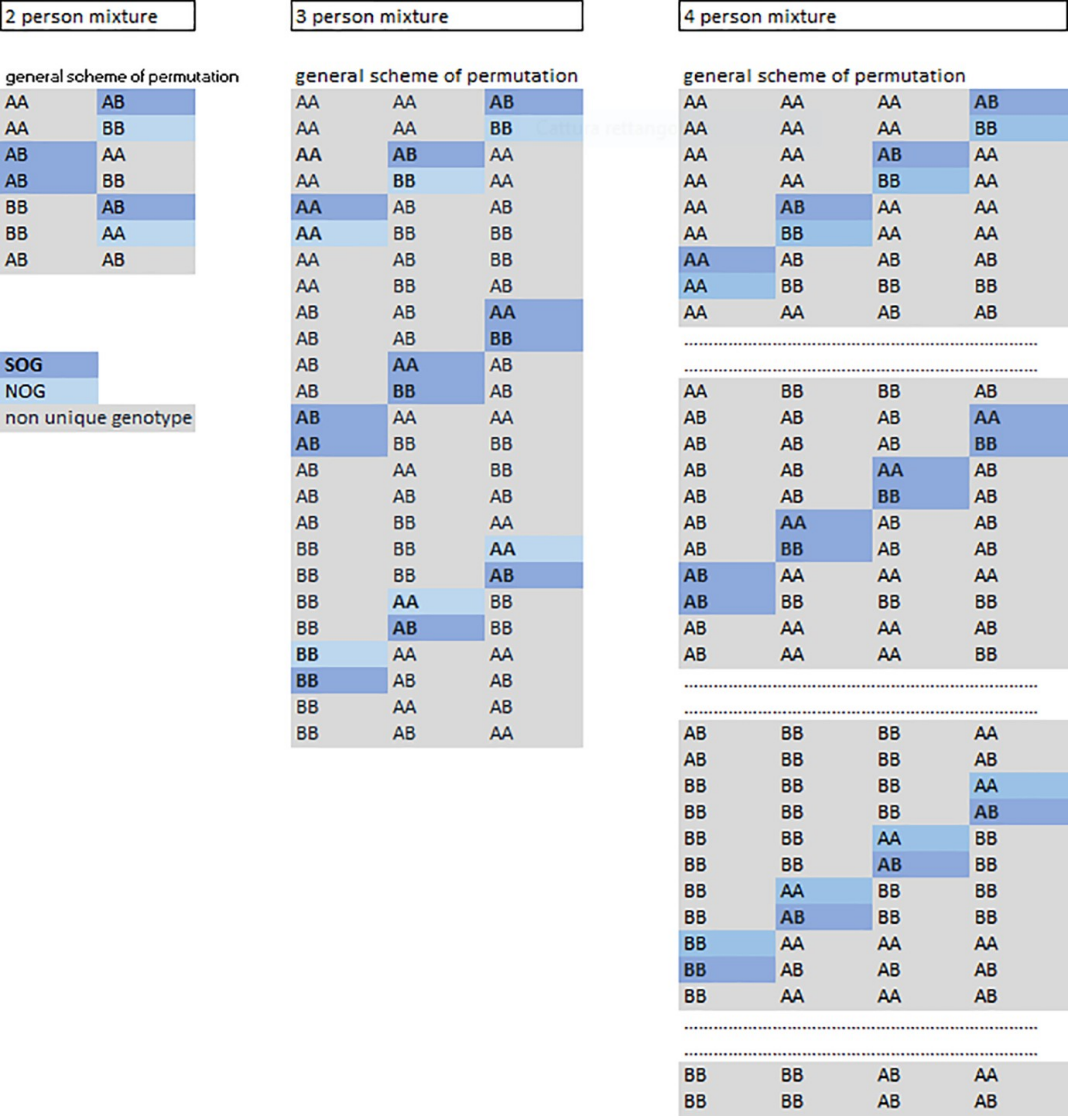
NOGs and SOGs at SNP mixtures. Genotypic matrices pertaining to a 2-person, 3-person and 4-person mixture are shown. Within each matrix, the network of unique genotypes is indicated with dark blue color (unique genotypes with simple-overlapping; SOCs) or light blue color background (unique genotypes with no overlapping; NOCs). The transition for two to three to four contributors and the elongation of the relevant matrix is entirely because of the introduction of various redundant combinations of genotypes, whereas the share of unique genotypes remains constant across the three matrices. To let us appreciate that the NOC/SOC network is the same, the 4PM scheme has been shortened by eliminating three groups of 15 permutations containing only redundant genotypes only (originally placed where the dotted lines are).

Interestingly, mixture matrices grow by accumulating redundant/overlapped combinations **and** by retaining the same NOGs/SOGs network (Fig 3).

NOGs/SOGs are of no use when no conditional hypothesis on the presence of a POI is formulated. At a matrix with no POI, NOGs/SOGs quantities are similar to all the other genotype quantities in such a way that the final mixture ratio equalizes (2PM = 0.5:0.5; 3PM = 0.33:0.33:0.33; 4PM = 0.25:0.25:0.25:0.25). Only once a POI is available, their unique genotypes align with one another and become discernible from the pool of unknown contributors. For every given POI there arise the following unique genotype alternatives: (POI unique genotypes in red typeface, non-POIs unique genotypes in blue):

POI AA: AA+(n-1)BB aut (n-1)AA+BB; *but also*: AA+(n-1)AB aut (n-1)AA+AB

POI AB: AB+ (n-1)AA aut (n-1)AB+AA *but also*: AB+(n-1)BB aut (n-1)AB + BB

POI BB: BB+(n-1)AA aut (n-1)BB+ AA *but also*: BB+(n-1)AB aut (n-1)BB+ AB

The availability of two combinations containing the same unique genotype generates uncertainty in the way to assign unique POI and unique non-POI quantities. However, within our nomenclature system some of these combinations (AB+BB; BB+AB) are **unrealistic** because they contradict the Q_a_>Q_b_ rule (there cannot be more ‘b’ than ‘a’ DNA molecules). And by a combinatorial exercise one can easily show that as long as matrices are built by the Q_a_>Q_b_ rule, unrealistic combinations appear in big number only when a false contributor is chosen as POI, whereas choosing a true contributor as POI will mostly intercept AA+AB combinations. There is therefore much sense of collecting unique genotype quantities from only:

POI AA: AA+(n-1)AB **and** (n-1)AA+AB

POI AB: AB+(n-1)AA **and** (n-1)AB+AA

POI BB: BB+(n-1)AA **and** (n-1)BB+AA

To intercept the underlying overall MR, a series of POIs and non-POIs unique genotypic amounts have to be collected from just one of these three combinations at each locus (the type of combination depending on the POI genotype), and the three datasets are separately summed together and a mixed ratio (MR_ex_) is calculated as follows:

Let us assume i to be the number of loci where POI = AA, j the number of loci where POI = AB, l the number of loci where POI = BB. Then the expected POI DNA amount is:

$$Q_{exPOI}=\sum_{1}^{i}QAA+\sum_{1}^{j}QAB+\sum_{1}^{l}QBB=\sum_{1}^{i}(Qa-Qb)+\sum_{1}^{j}2Qb+\sum_{1}^{l}Qb$$

The expected amount for the unknowns (Us) is:

$$(Q_{exU}|POI)=\sum_{1}^{i}2Qb+\sum_{1}^{j}(Qa-Qb)+\sum_{1}^{l}Qa$$

And the expected mix ratio is:

$$MR_{ex}=Q_{exPOI}/[Q_{exPOI}+(Q_{exU}|POI)];(Q_{exU}|POI)/[(Q_{exPOI}+(Q_{exU}|POI)];$$

For details on how to implement this procedure on a spreadsheet, the reader should refer to a practical example (ex_MR_calc_115_XEN46_P1P2P3RND.xlsx) included in the S1 File attached to this article.

The expected MR has the following properties: if a true contributor is chosen as a POI, all POI NOG-SOG DNA amounts (collected from all loci) will cluster at /around the same MR; if the POI is a false contributor, all SOG/NOG combinations will return balanced ratios (e.g., 2PM with 0.5: 0.5 MR; 3PM with 0.33:0.33:0.33 MR; 4PM with 0.25:0.25:0.25:0.25 MR). True contributors within a balanced mixture behave as if they were false contributors. If more than one POI is available, the respective SOGs/NOGs should be extracted from the permutations that contain them all. To conclude, MR_ex_ is the **expected value** for a series of (1 - χ^2^) tests to set at every permutation state of each locus of an SNP protocol.

#### 2.3.4. Setting the (1-χ^2^) statistics

It is now necessary to distribute the distinctive probabilities of existence to each permutation state of a mixture. This will be accomplished by setting a (1 - χ^2^) within every permutation.

The rationale underlying the use of the (1 - χ^2^) test is the following:

- Quantities originating from unique genotypes (MR_ex_) invariably reflect the original ratio established by the true contributors.
- Quantities assigned by the autosomal Mendelian rules to all permutation states (we will call them ‘MR_obs_’) will comply with the MR_ex_ only if the corresponding genotypes exactly coincide with those originally contributed by the true mixture founders; in all other cases, the MR values will be sparse.
- Assuming MR_ex_ as the expected value and all other “Mendelian” MR_obs_ as the individual observed values and setting a (1 - χ^2^) test will give each permutation state a distinctive probability based on the DNA apportioning.

As previously stated, there is just one MR_ex_ but numerous observed MR_obs_. In a previous chapter (“Giving MRs to all permutation states”) we have given rules to calculate them. In the S1 File attached to this article we will apply these rules, with relevant algebra, to the case of 2PM and 3PM.

#### 2.3.5. Calculating quantitative likelihoods by three statistical indexes

Our quantitative scheme of analysis now contains three well-established statistical indices: the (1 - χ^2^) MR value, the h index (the minor peak read divided by the major peak read), and the population genotype frequency. The three values reflect several independent properties of the mixtures: the MR pertains to the specific trace, the h index is a Mendelian autosomal property, and the genotype frequency is a population statistic. The three indices repeat themselves with different values at each permutation state; they can multiply at this level and give each state a synthetic probability of existence. All products of all permutation states are summed to give the value of any likelihood of interest.

For example, the probability of a mixed evidence E{a;b} under the prior assumption of two contributors and one POI with genotype AB and the following experimental data:

(PE{a;b} |K{a;b}; n = 2;η = 1; f_a_ = 0.445; f_b_ = 0.555; Q_a_ = 739; Q_b_ = 704; MR_ex_ 0.94;0.06)

is a likelihoods that allows for 3 permutation states: (AB+AB); (AB+BB); (AB+AA). The value of the three permutations is:

P(AB+AB): Mendelian index = P(GT) = 1*(2*0.455*0.555); h index = 0.952; (1-χ^2^;MR_obs_0.9:0.1; MR_exp_ 0.9:0.1) = 1; then: 0.494*0.952*1 = 0.47.

P(AB+BB) is: Mendelian index = P(GT) = 1* (0.555^2^); h index = [(2*704)/3 /739] = 0.635; (1-χ^2^; MR_obs_ 0.7:0,3; MR_exp_ 0.9:0.1) = 0.169; then: 0.308*0.635*0.169 = 0.03

P(AB+AA) is: Mendelian index = P(GT) = 1* (0.445^2^); h index = 1; (1-χ^2^; MR_obs_ 0.98:0.02; MR_exp_ 0.9:0.1) = 0.924; then: 0.198*1*0.924 = 0.18

Therefore, the value of the likelihood is 0.47 + 0.03 + 0.18 = 0.687.

We report formulae for calculating the simplest likelihoods in the S1 File attached to this paper. These formulae are also implemented within three calculation worksheets available as S1 File.

### 2.4. Testing datasets

To test the performance of our calculation methods we downloaded a collection of evidence data (massive parallel sequencing read data referring to 140 loci forensic SNPs) and reference data (three ‘true contributors; or individuals P1,P3,P2 typed for the same 140 SNPs) available at the open-source EuroForMix website http://www.EuroForMix.com/?q=data). These mixtures were prepared in vitro [36] by blending DNA aliquots of P2,P3,P2 so as to reproduce a series of 2PMs (individuals P1 and P2; ratios 1:1, 1:3, 1:9; 3:1; 9:1 and 3PMs (individuals P1, P3, P2; ratios: 1:1:1; 1:1:5; 1:5:1; 1:5:5; 5:1:1; 5:1:5 and 5:5:1). A selection of all available reference data (the XEN13_a and the XEN46_a subsets) was considered for our statistical analysis and encoded according to our alphabetical nomenclature. The original 140 loci in this evidence were reduced to 133 loci by eliminating incomplete typing data records. An array of European gene frequencies available on the EuroForMix website was used. To complete our inferential scheme, we obtained 40 more reference data of false contributors to use as POI, provided graciously by Professor Andreas Tillman (Department of Forensic Genetics and Forensic Toxicology, National Board of Forensic Medicine, Linköping, Sweden).

Each group of evidentiary data was analysed by adopting our 2-person, 3-person and 4 person likelihood calculation routines, regardless of the fact that the original evidence would originate from a two-person or a three person mix. Our MRs and LRs were compared to those generated by the EuroForMix shareware (version 3.0.3, developer: Dr Ø. Bleka).

It is here appropriate to notice that both the EuroForMix shareware and our Excel worksheets do not allow to compute LRs referring to mixture models based on more than 4 contributors.

## 3. Results

### 3.1. MRs from XEN 13a /XEN 46a, three true contributors as POI and a variable amount of unknowns

**Fig 4:**
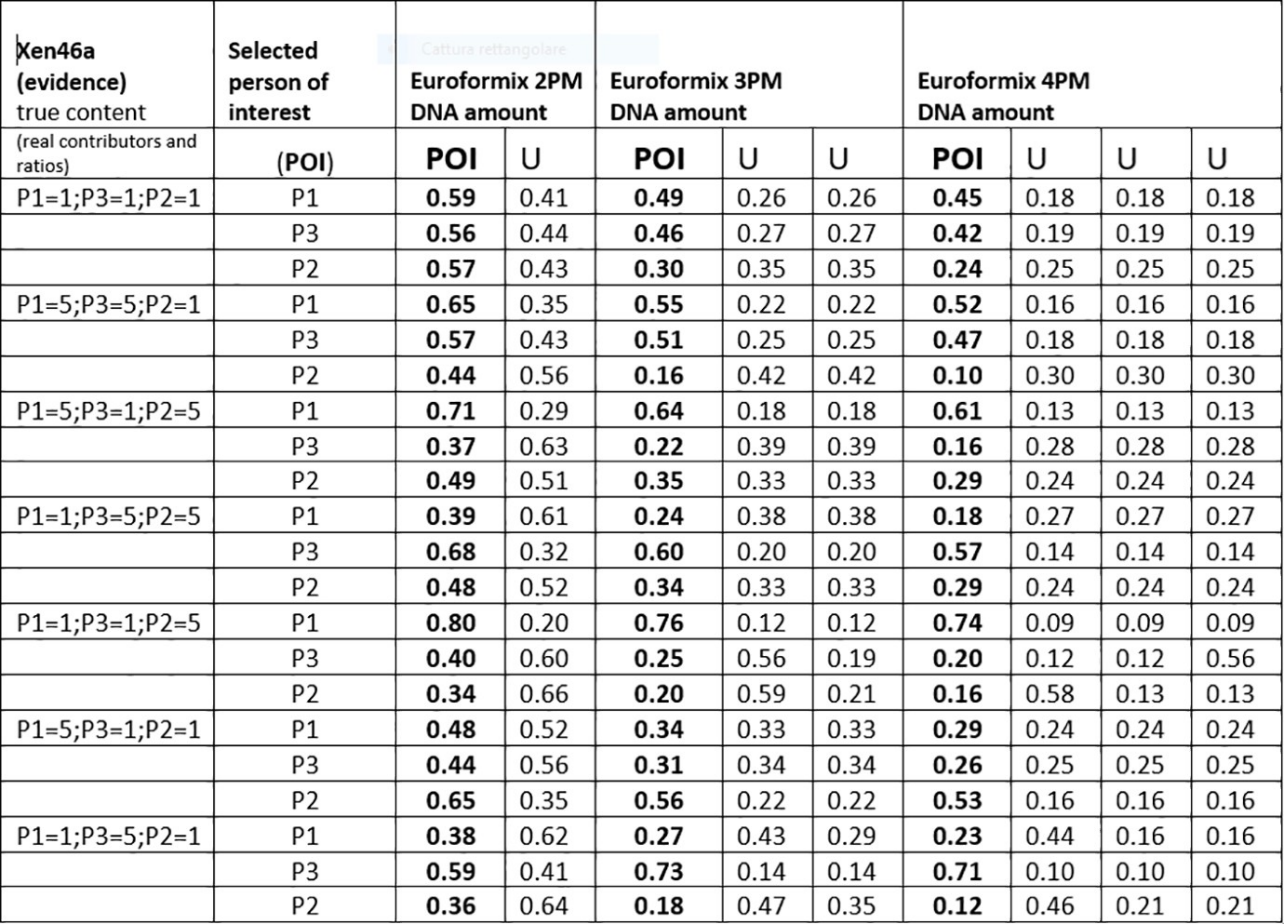
Amount of DNA assigned to a true contributor chosen as the person of interest (POI) and to the unknown contributors (U) at experimental evidence built by mixing three contributors (P1, P3, and P2) (dataset: XEN46a). The DNA amounts are calculated according to the 2-person, 3-person, and 4-person schemes. The EuroForMix software is used.

**Fig 5:**
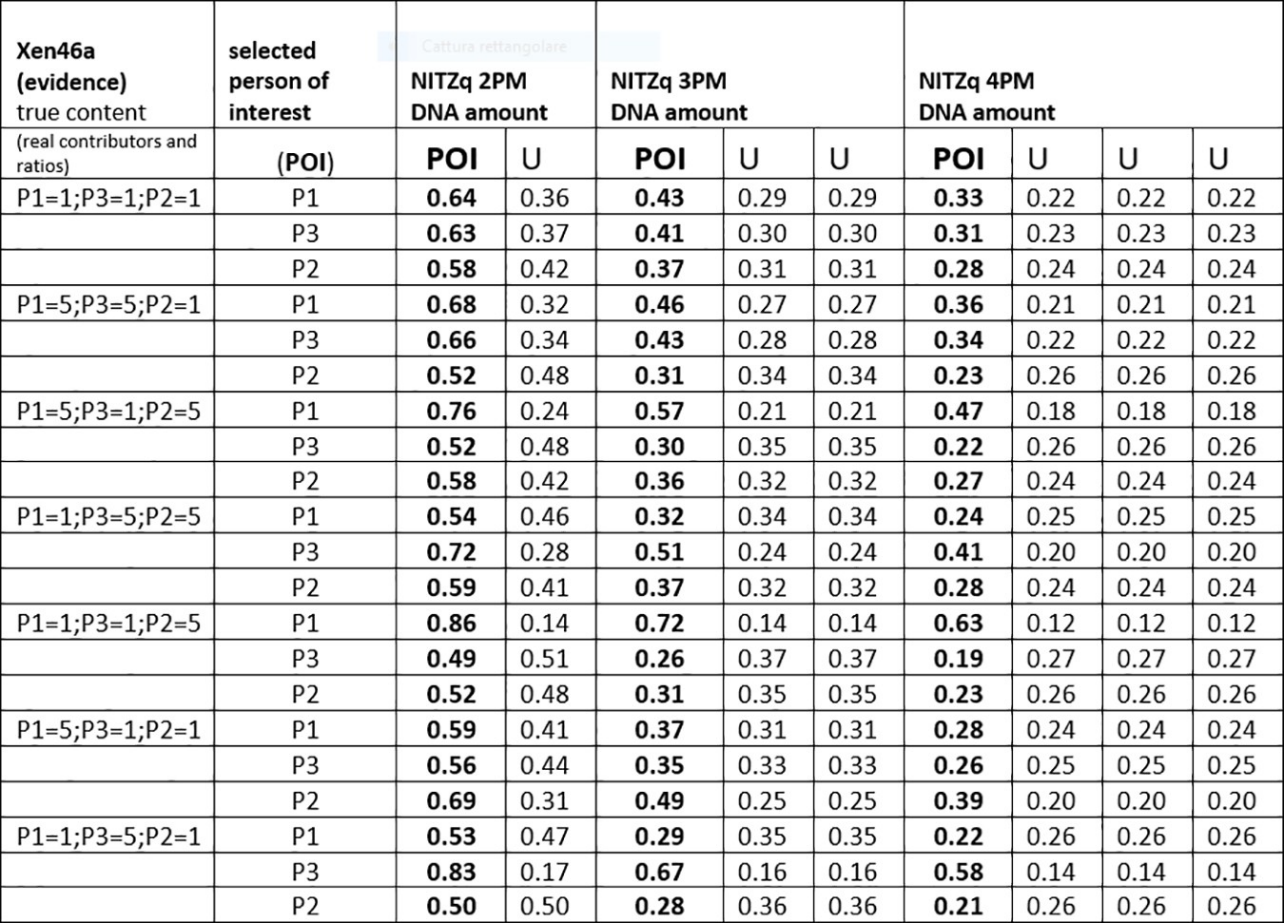
Amount of DNA assigned to a true contributor chosen as the person of interest (POI) and to the unknown contributors (U) at experimental evidence built by mixing three contributors (P1, P3, and P2) (dataset: XEN46a). The DNA amounts are calculated according to the 2-person, 3-person, and 4-person schemes. The NITZq worksheets are used.

**Fig 6:**
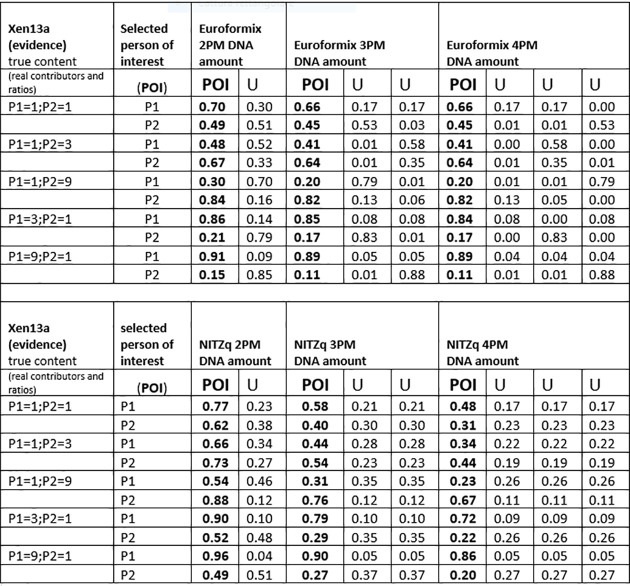
Amount of DNA assigned to a true contributor chosen as the person of interest (POI) and to the unknown contributors (U) at experimental evidence built by mixing two contributors (P1 and P2; XEN13a). The DNA amounts are calculated according to the 2-person, 3-person, and 4-person schemes. The EuroForMix software and the NITZq worksheets are separately used.

Figs 4–6 show twelve series of mix ratio predictions as they come out from our NOGs/SOGs-based method and from the EuroForMix software. We used the three NITZq worksheets to calculate the NOGs/SOGs MR, and will refer to the NOGs/SOGs MR as the “NITZq MR.” We used EuroForMix version 3.0.3. Evidentiary datasets were XEN46a (a mix made of three contributors P1, P3, P2 mixing at different ratios of 1:1:1, 5:5:1, 5:1:5, 1:5:5, 5:1:1, 1:1:5, 1:5:1; quoted in the order P1, P3, P2) and Xen13a (made of two contributors P1 and P2 blending at ratios 1:1;1:3;1:9; 3:1; 9:1; first quoted quantity is P1). The P1, P3, and P2 were individually used as the POI along with one, two, and three additional unknown (U) contributors (KU; KUU; KUUU; here the synthetic notation K stands for “POI”). The POI is here a “true” contributor—a person whose DNA had been used to assemble the mixture evidence—his genotypes having been downloaded from the EuroForMix website. Only one of the three hypotheses on the number of contributors used to interrogate each evidentiary example was assumed to be “true” based on what was declared on the EuroForMix website. The other predictions were made by assuming an incorrect number of contributors. These data shows that NITZq quantitative predictions on the POI DNA amount are similar to those issued by the EuroForMix software—whereas predictions on unknown contributors operated by the two methods may occasionally diverge. In principle, NITZq always flattens the unknown MR proportions to exactly the same value; EuroForMix occasionally assigns differential values, other times distributes just one value to all unknowns. Predicting quantities under an incorrect assumption about the true number of contributors has a limited effect on the share assigned to the POI—in the range of two to four contributors. But in principle, expanding the contributors’ number shrinks the quantitative proportion left to a true POI. Both NITZq and EuroForMix sometimes diverged from the mix ratio originally declared by the authors who built the experimental mixtures [36] presumably due to experimental dilution errors and, occasionally, owing to clerical errors committed by the data producers (as acknowledged by the authors [36] at XEN46 ratio 1:1:5 P1 (and not P2) is in reality the predominant contributor; at XEN46 ratio 5:1:1 P2 (and not P1) is the predominant DNA).

### 3.2. MRs, with false contributors in the role of POI

**Fig 7:**
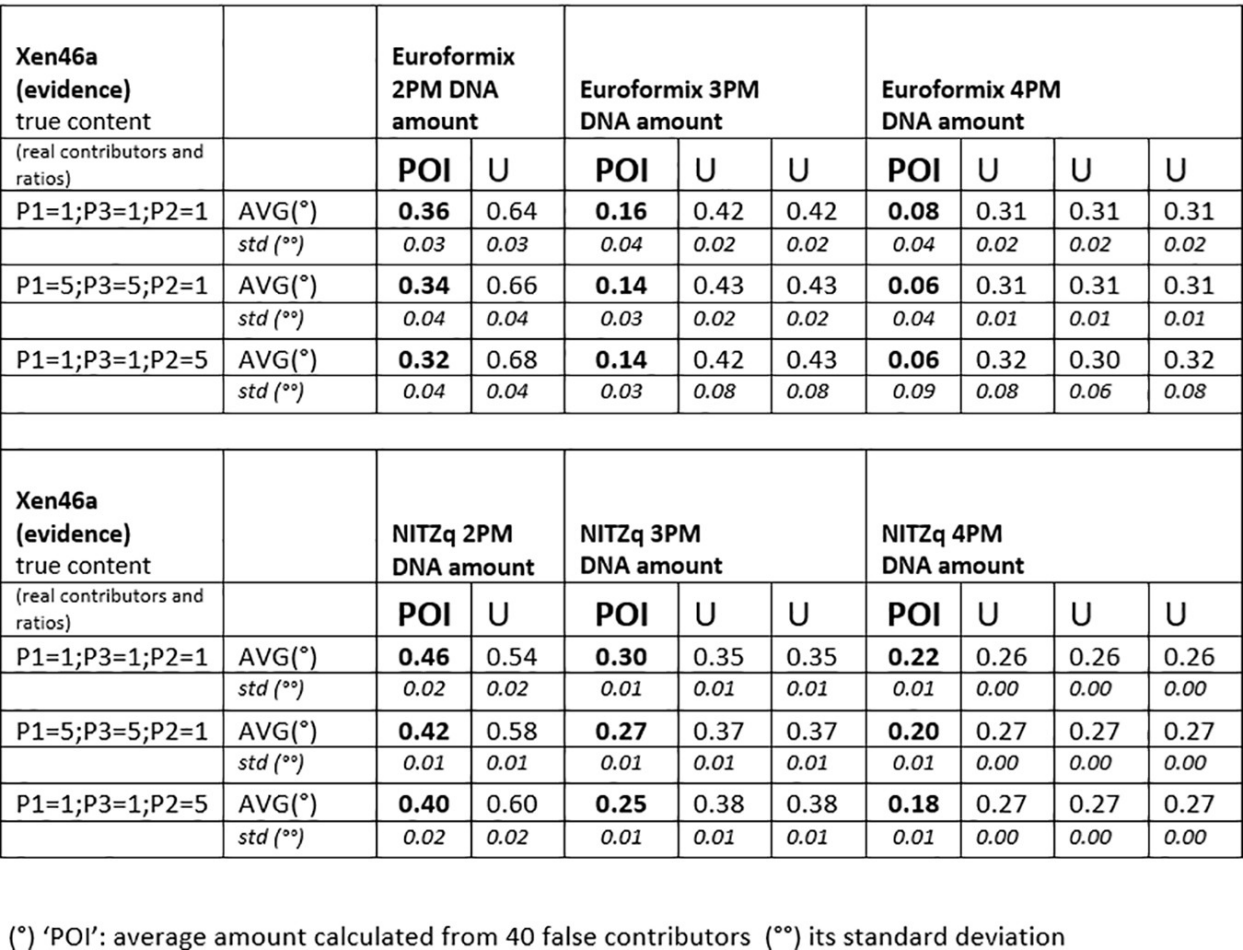
The average amount of DNA assigned to a cohort of 40 false contributors placed (one at the time) in the role of POI at three-person-based experimental evidence (XEN46a). The 40 contributors returned nearly the same DNA amount fraction with very little standard deviation. Thus, the 40 values were averaged, and the average value is shown in the figure, instead of individual values. POI, person of interest; U, unknown contributor.

**Fig 8:**
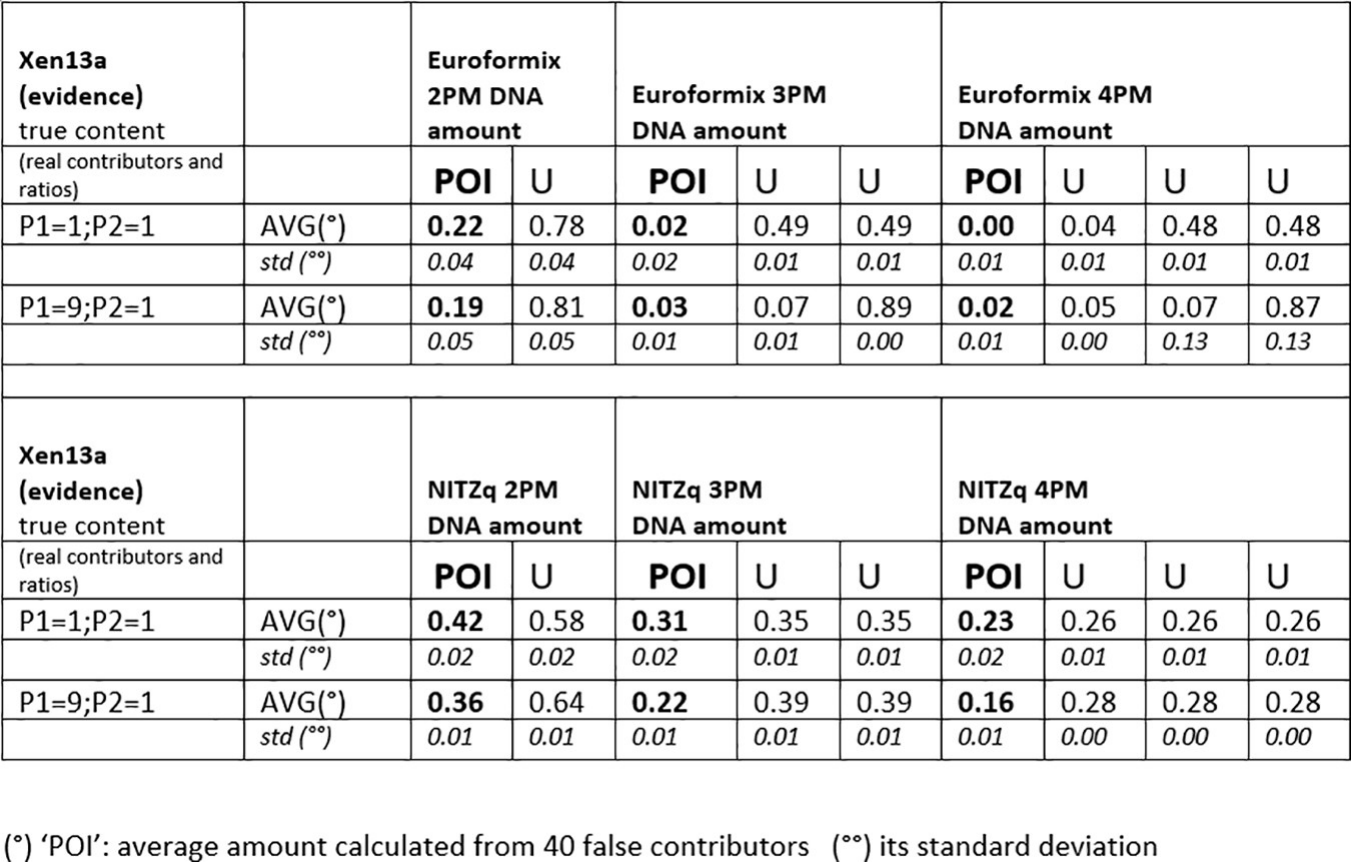
The amount of DNA assigned to a cohort of 40 false contributors placed (one at the time) in the role of POI at two-person-based experimental evidence (XEN13a). The 40 contributors returned nearly the same DNA amount fraction with very little standard deviation. Thus, the 40 values were averaged, and the average value is shown in the figure, instead of individual values. POI, person of interest; U, unknown contributor.

Another series of MR predictions were produced by replacing the “true” P1, P3, and P2 with false contributors in the role of POI. False contributors were taken one by one among a population of 40 unrelated Swedes (data provided by Prof. Tillmar). The predictions followed the same scheme as before, but for simplicity, each POI was matched to a fewer evidentiary dataset (XEN13, ratios: 1:1, 1:9; XEN46a ratios: 1:1:1, 1:5:5, 1:1:5). With both NITZq and EuroForMix, the quantitative fractions predicted for each of the 40 POIs were so close to one another that they could be conveniently averaged with little standard deviation, as shown in Figs 7 and 8. These figures reflect the different approaches of the two methods: EuroForMix seeks the best fitting quantitative proportions in numerous random simulations, whereas NITZq singles out values tending to 1/n, which is the usual result of a free permutation (with no available POI). We envision that the 1/n value is eventually reached by predicting MRs over a very large number of loci. Increasing the number of contributors has the effect of halving the quantitative fraction assigned by EuroForMix. NITZq moves in a similar direction, but the decrease in the POI quantitative proportion has its limit at 1/n. Matching the same POIs to balanced or unbalanced mixtures has little effect on the predicted proportions.

### 3.3. LR values from true contributors as POI

**Fig 9:**
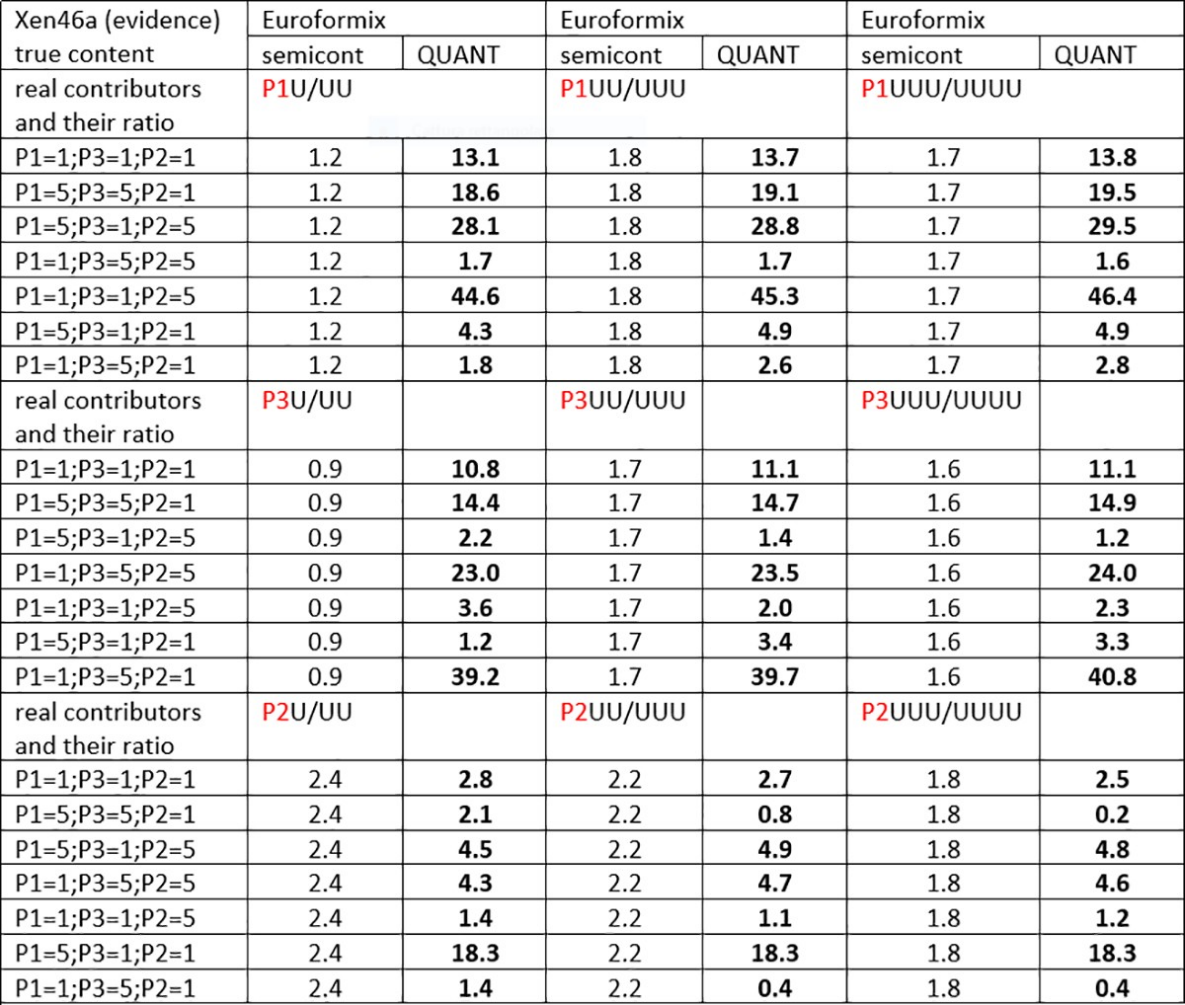
Log10(LRs) pertaining to three true contributors matched (one by one) as POI to an experimental three-person mixture (XEN46a). EuroForMix calculations are shown. The original likelihood ratios are built by placing a POI and one to three unknown contributors in the numerator (for example P1 + U; P1 + UU; P1 + UUU) and all unknown contributors in the denominator (UU; UUU; UUUU).

**Fig 10:**
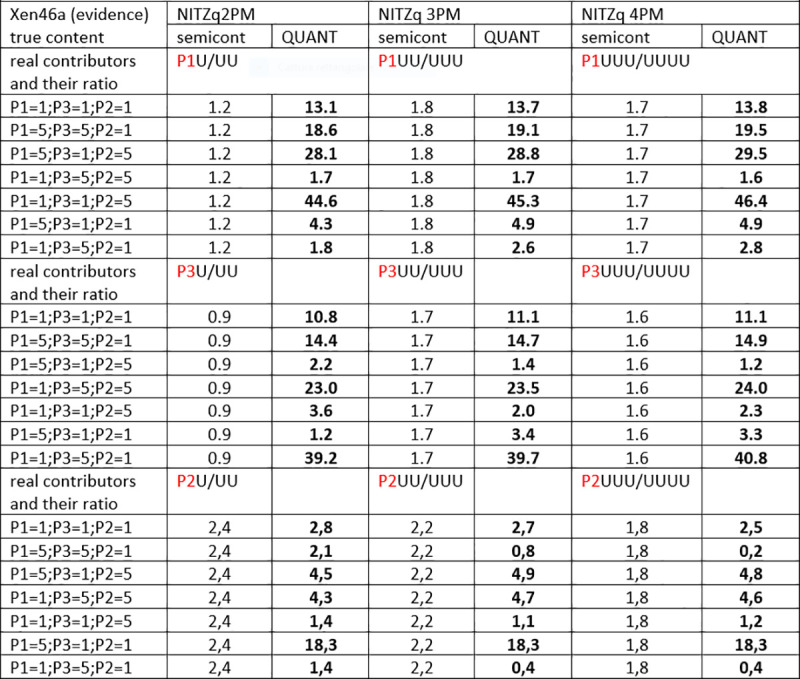
Log10(LRs) pertaining to three true contributors matched (one by one) as POI to an experimental three-person mixture (XEN46a). NITZq calculations are shown. The original likelihood ratios are built by placing a POI and one to three unknown contributors in the numerator (for example P1 + U; P1 + UU; P1 + UUU) and all unknown contributors in the denominator (UU; UUU; UUUU).

**Fig 11:**
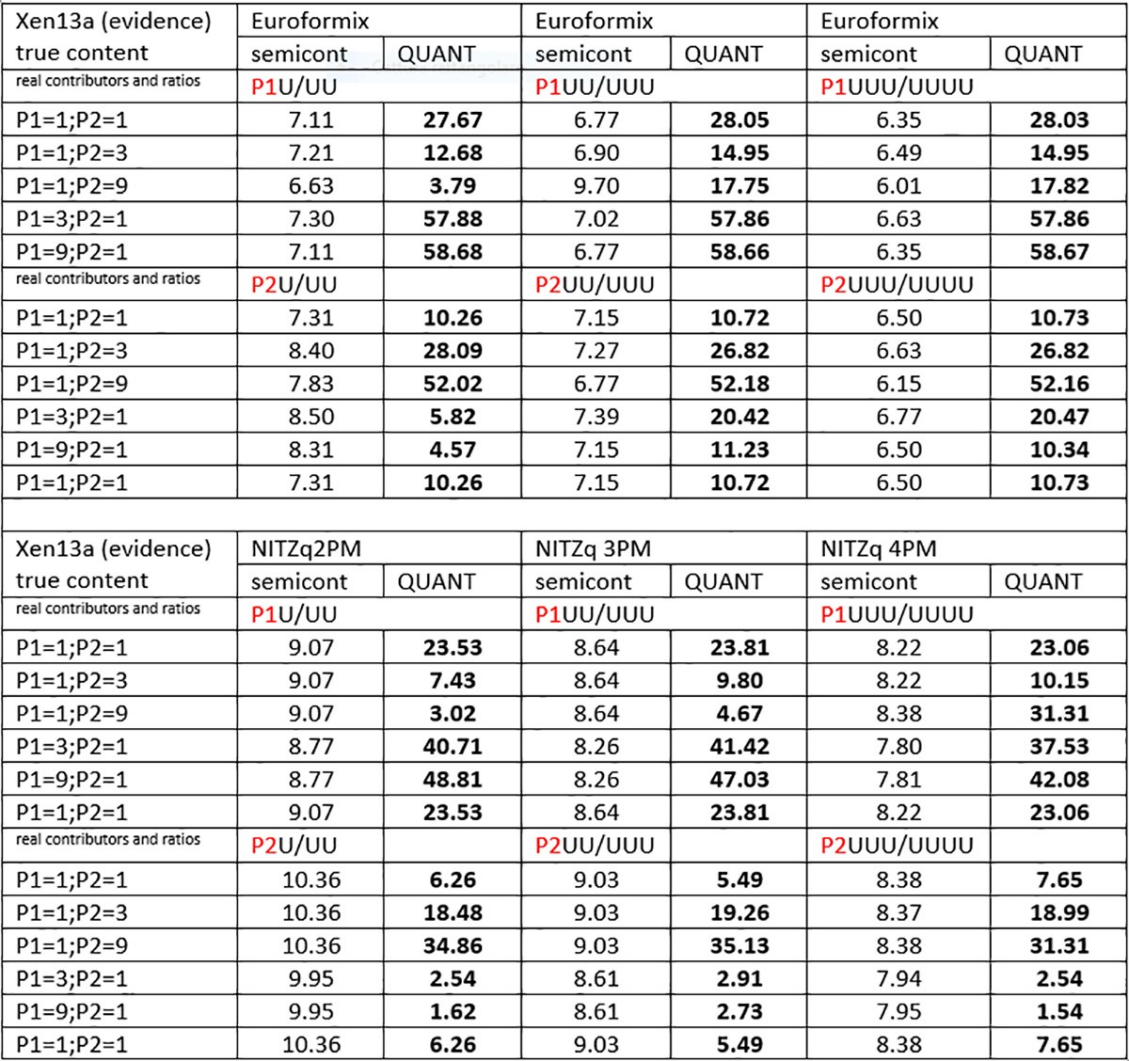
Log10(LRs) pertaining to three true contributors matched (one by one) as POI to an experimental two-person mixture (XEN13a). EuroForMix and NITZq calculations are here shown in comparison. The original likelihood ratios are built by placing a POI and one to three unknown contributors in the numerator (for example P1 + U; P1 + UU; P1 + UUU) and all unknown contributors in the denominator (UU; UUU; UUUU).

The Log10(LR) values calculated from the two tutorial datasets are shown in Figs 9–11. Here again, we used our three NITZq worksheets to produce our LR data; thus, we will henceforward refer to our quantitative LRs as to the NITZq LRs. To produce these data, we set NITZq to call the biallelic evidence E{a;b} every time the minor allele read would be greater than or equal to the major read multiplied by 0.05 and to call the monoallelic E{a} below this value. EuroForMix version 3.0.3 calculations were based on an absolute minor allele calling threshold of five reads and a P(D) = 0.05. The semi-continuous LRs are of comparable value if the two systems of calculation are set to call the same number of homozygous genotypes within the whole locus evidence. Betting on the wrong number of contributors does not significantly affect the semi-continuous LRs of either system.

Both NITZq and EuroForMix LRs return a comfortably high WoE in tests where the POIs are true contributors and match the evidence. However, NITZq underestimates LRs by one to sometimes ten orders of magnitude (or bans), compared to EuroForMix, every time the POI amounts predominate within the evidence. Although both methods give very high LRs, this circumstance prompted us to investigate further. Therefore, we selected experiments with unbalanced evidence and extracted all individual locus-specific LRs obtained from both computation methods and compared them to the corresponding single–source LR values (1/P(GT); or 1 divided by the genotype frequency of the POI).

To provide a typical example, the unbalanced evidence XEN46 1:1:5 (originally made up by mixing five parts of the contributor P1 DNA with one part P2 and one part P3; then an unbalanced 3PM) was compared to the three true contributors as POI. The locus-specific LRs were calculated under two, three, and four contributors’ prior hypotheses (the 3PM being the true hypothesis), then copied into a worksheet. The NITZq and EuroForMix datasets were then separately compared to a series of 1/P(GT) data corresponding to each of the 133 loci, using the relevant pair of gene frequencies as data sources. All results and comparisons about this example were collected in an Excel file (LR_over_1_GT_at_115_xen_46_P1P2P3.xlsx) to be found in the S1 File of this article

As a result of this comparison and concerning the content of this documental file, we can state that:

1. When a quantitatively predominant true contributor is used as POI (for example P1 at XEN46 1.1:5) EuroForMix version 3.0.3 returns LRs that exceed the corresponding 1/P(GT) values at numerous loci of the protocol (56 out of 133 loci in the 3PM model of analysis, 63/133 in the 4PM model). Under the same circumstances, the NITZq LRs never exceed those ceiling values. As it has been repeatedly asserted that the WoE against a suspect based on a mixture model can never be stronger than the WoE obtained by a single-source DNA profile [27, 50], it can be concluded that the EuroForMix WoE predictions in these cases are seriously overestimated.
2. When the POI (P3 and P2 in Xen46, a 1.1.5) enters the mixture evidence with proportionally lower DNA amount than other contributors, the two computational methods return nearly the same WoE, and neither EuroForMix version 3.3 nor NITZq LRs overcome the 1/P(GT) ceiling.

These two facts are proof-of-principle that EuroForMix may occasionally perform better than NITZq (for example, XEN46a 1:1:5 with POI = P1: EuroForMix πLR = 1.7E + 45; NITZq πLR = 1.2E + 37) only at the cost of violating the fundamental rule of forensic inference: LR_mixture_ < 1/(P(GT)). Because of these findings, we can conclude that NITZq predictions are more reliable and realistic than those issued by EuroForMix, which sets a key point in the favor of our mathematical method.

### 3.4. LR values for false contributors as POI

**Fig 12:**
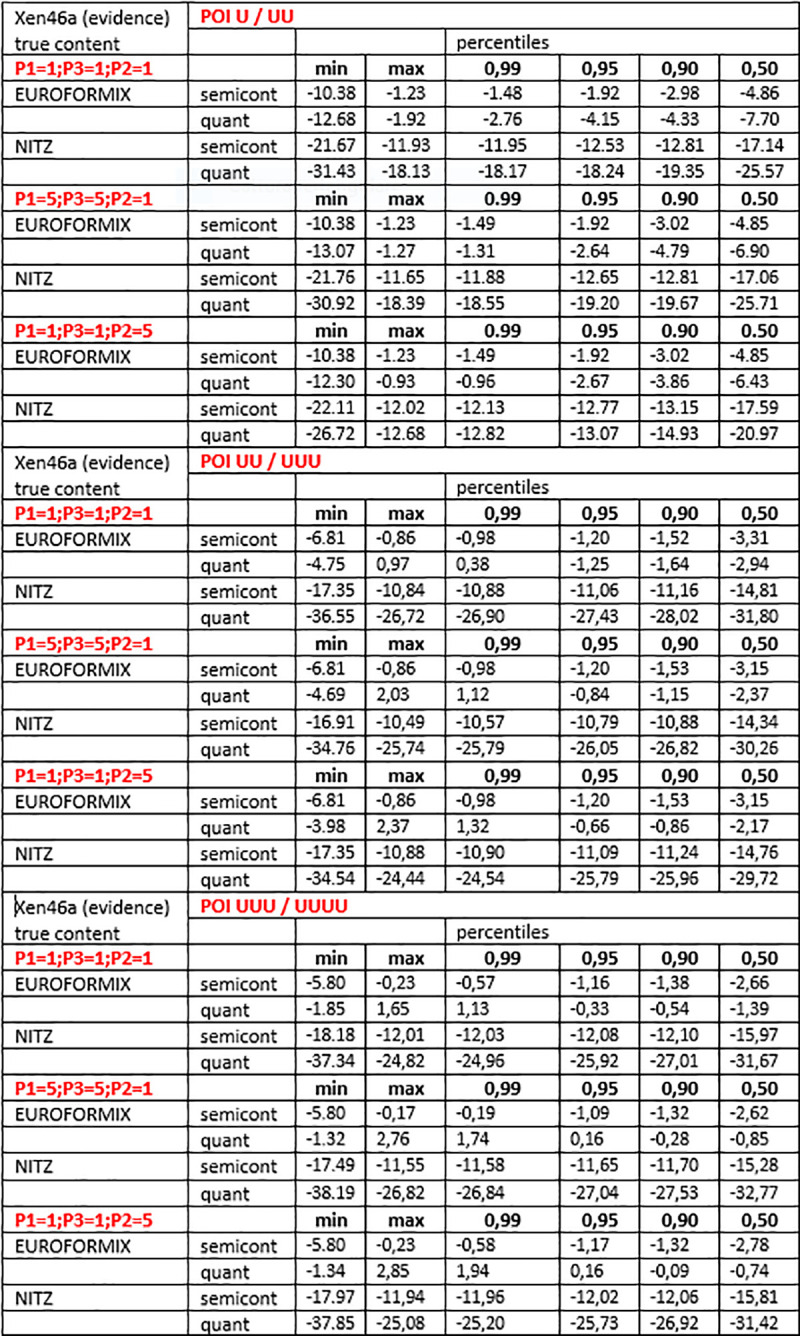
Log10(LRs) pertaining to 40 false contributors matched (one by one) as POI to three different versions of an experimental three-person mixture (XEN46a; true contributors P1,P3,P2 with quantitative ratio: 1:1:1, 5:5:1, 1:1:5). EuroForMix and NITZq calculations are shown in comparison. POI U/UU; POI UU/UUU and POI UUU/UUUU refer to schemes of likelihood ratios with 2,3, and 4 contributors, where the numerator differs from the denominator by the presence of just one person of interest.

**Fig 13:**
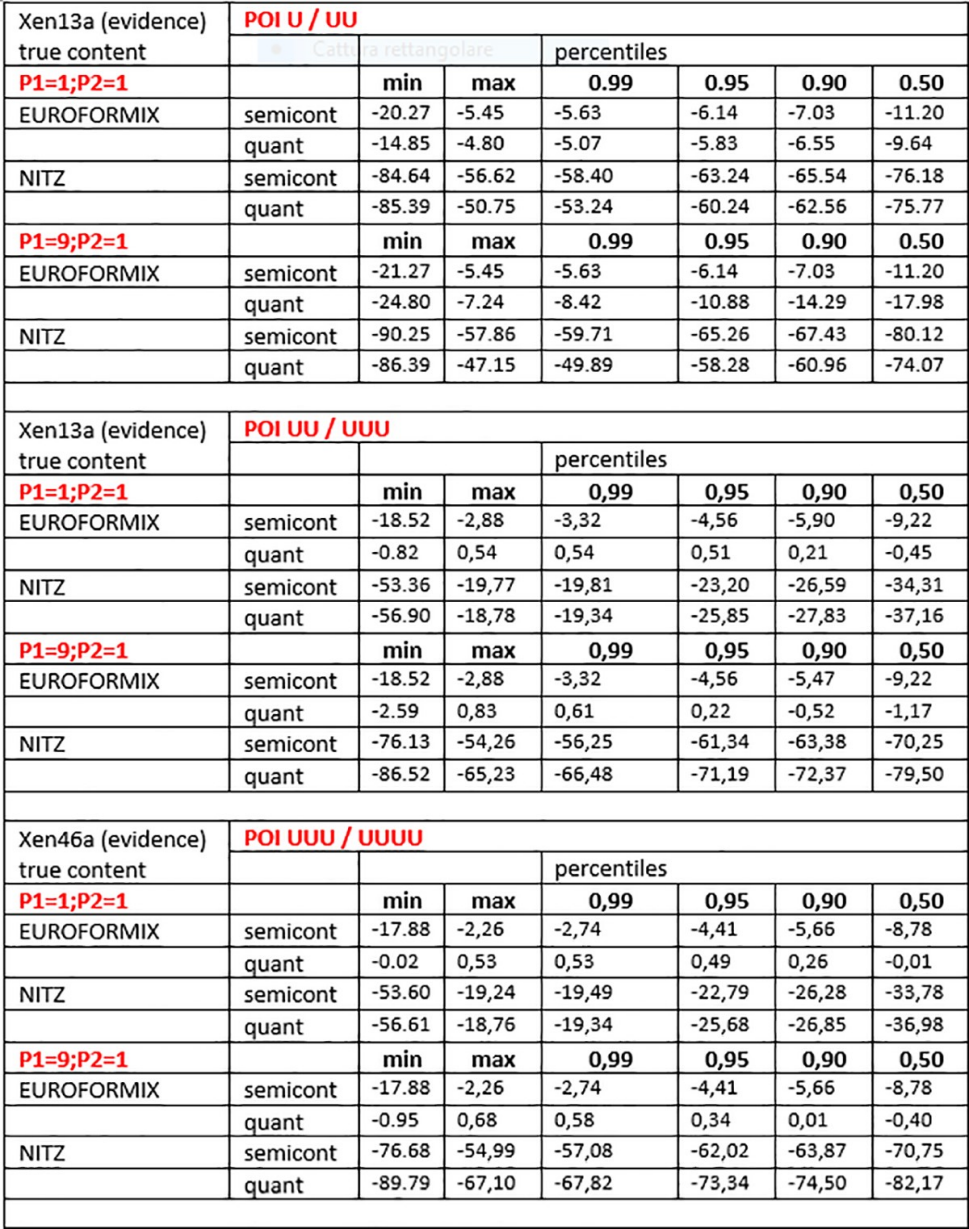
Log10(LRs) pertaining to 40 false contributors matched (one by one) as POI to two different versions of an experimental two-person mixture (XEN13a; true contributors P1,P2 with quantitative ratio: 1:1, 9:1). EuroForMix and NITZq calculations are shown in comparison. POI U/UU; POI UU/UUU and POI UUU/UUUU refer to schemes of likelihood ratios with 2,3, and 4 contributors, where the numerator differs from the denominator by the presence of just one person of interest.

These datasets were produced using the same analysis scheme detailed in the previous section. An excerpt of the whole data showing the maximum and minimum values of NITZq and EuroForMix LRs, along with the internal percentile distribution, is shown in Figs 12 and 13 (log10(LR) are shown). It is easily noticed that NITZq gives much lower LRs to false contributors, thus ruling out type-two errors (false inclusions) more efficiently than EuroForMix. This is essentially because of three issues:

- NITZq is highly efficient in spotting the homozygous evidence, owing to the dynamic threshold (based on the minor vs. major read ratio) used to call the minor-read allele.
- NITZq allows for more exclusion cases to emerge, with evidence E{a} either facing a POI = AB or a POI = BB.
- NITZq deals with these two classes of exclusion using algebra, according to Eqs 6–9; this results in low ((E{a} vs. K = AB) or very low (E{a} vs. K = BB) LR values.

### 3.5. Correlation between MR and LR values, with true contributors as POI

The LR vs. MR relationship was investigated at both the XEN46a and XEN13a evidence by matching P1, P2, and P3 (the “true” contributors) as POI to variously imbalanced mixtures. For simplicity, the LR values were calculated by adopting the true number of contributors (at XEN13a: KU/UU; at XEN46a: KUU/UUU). Then, the Log10(LR_quant_) minus the Log10(LR_semicont_) value was paired to the quantitative fraction predicted for each POI. Pairs of values were plotted together, regardless of the difference in genetic profiles originating from the data points.

**Fig 14:**
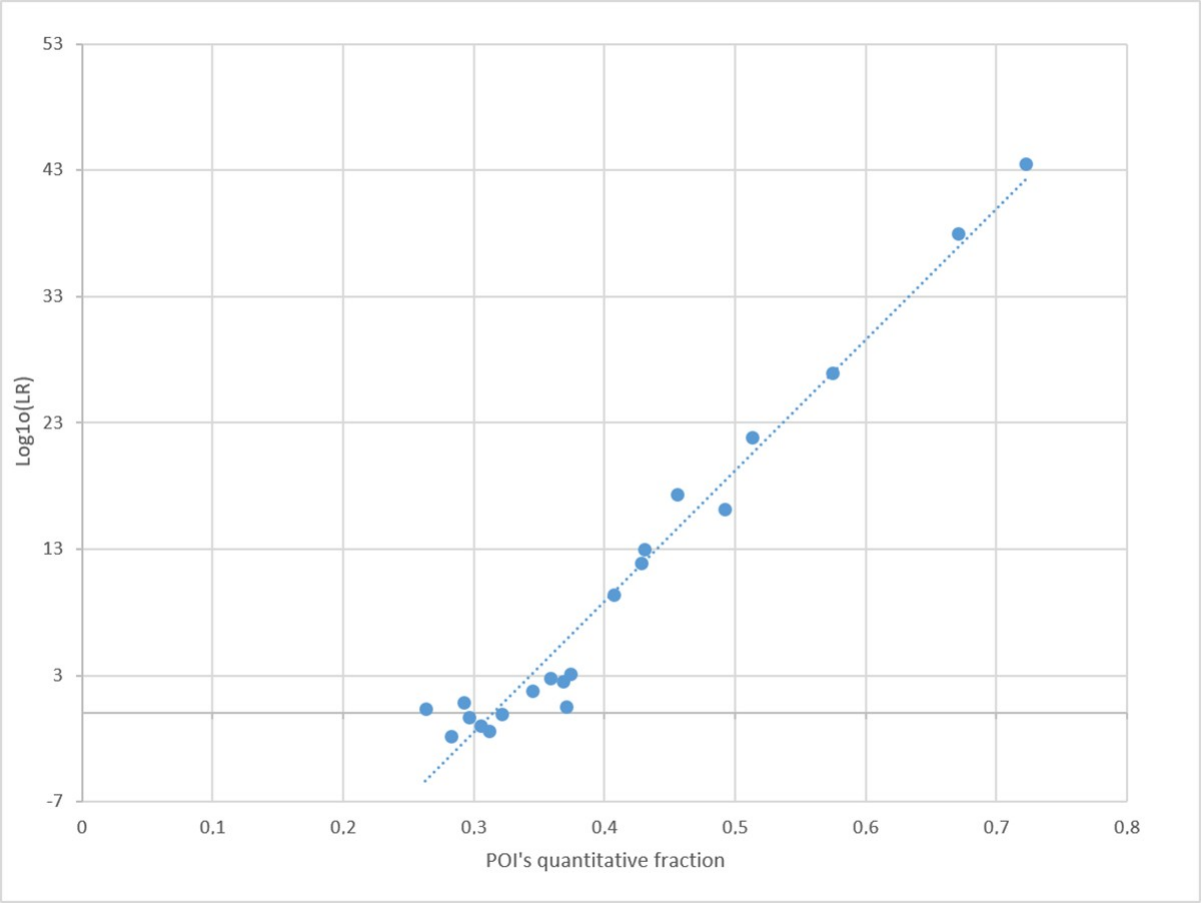
Plots of POI’s quantitative fraction vs POI’s Log10(LR) value in a series of true contributors’ tests. The evidence is XEN46_a, and POIs are P1, P2, and P3 (all downloaded from the EuroForMix website). A NITZq 3PM worksheet was used to produce all pairs of results.

**Fig 15:**
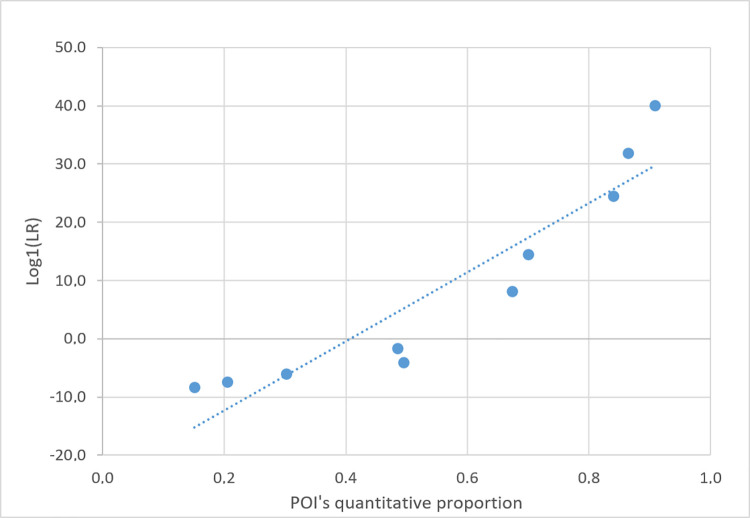
Plots of POI’s quantitative fraction vs POI’s Log10(LR) value in a series of true contributors’ tests. The evidence is XEN13_a, and POIs are P1 and P2 (all downloaded from the EuroForMix website). A NITZq 2PM worksheet was used to produce all pairs of results.

We found that when the true contributors are chosen as POI, a direct proportional relationship between the POI MR proportion and the Log10(LR) value emerges from the NITZq-generated series of data (Figs 14 and 15). The higher the MR fraction of the POI, the higher the LR value. This relationship holds even when an incorrect number of contributors is used to calculate the LRs.

### 3.6. MR and LR relationship–with false contributors as POI

**Fig 16:**
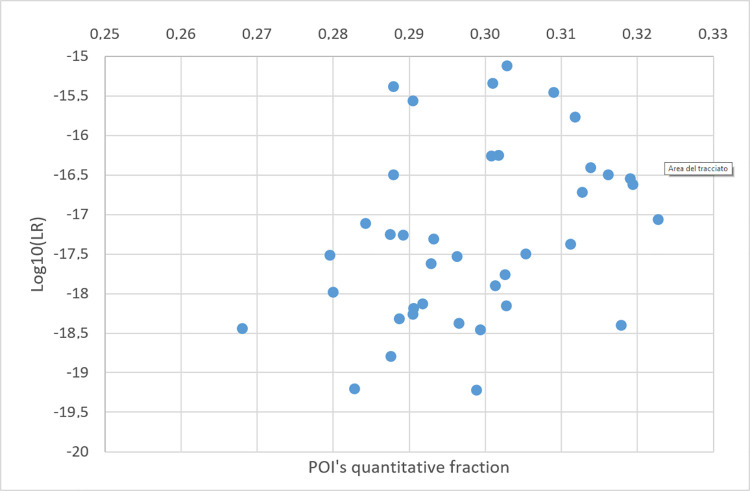
Plots of POI’s quantitative fraction vs POI’s Log10(LR) value in a series of false contributors’ tests. The evidence is XEN46_a ratio of 1:1:1, and POIs are 40 unrelated individuals. A NITZq 3PM worksheet was used to produce all pairs of results.

**Fig 17:**
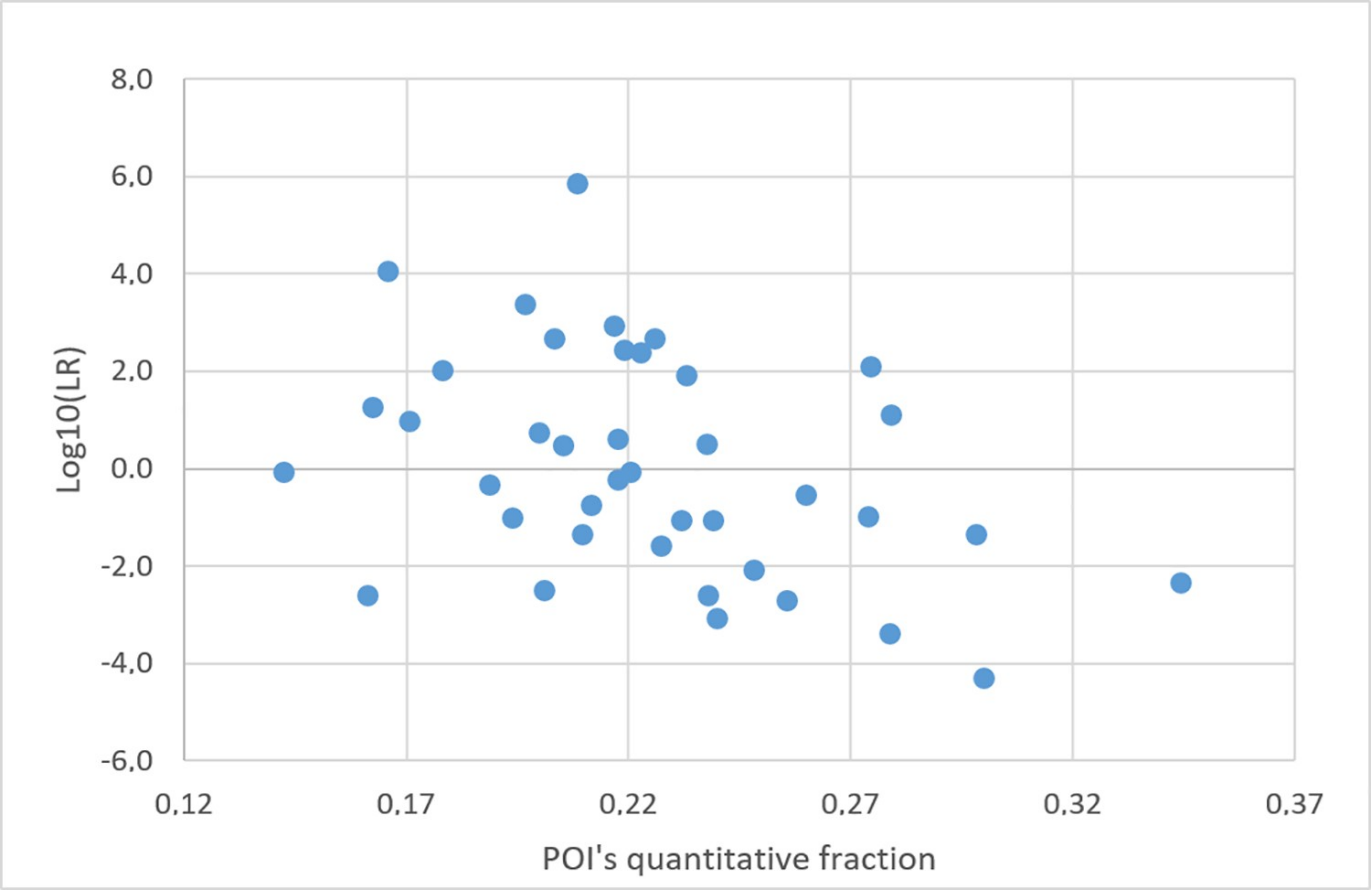
Plots of POI’s quantitative fraction vs POI’s Log10(LR) value in a series of false contributors’ tests. The evidence is XEN13_a ratio 1:1, and POIs are 40 unrelated individuals. A NITZq 2PM worksheet was used to produce all pairs of results.

This relationship was investigated using the same analysis scheme adopted in the previous section. Forty false contributors were chosen one at the time as the POI in the following schemes of analyses: XEN46a ratio 1.1:1, KUU/UUU; XEN13a ratio 1.1, KU/UU. In the false contributors’ tests, no coherent relationship was found between MR and the LR (Figs 16 and 17).

### 3.7. Similarities and differences between EuroForMix and NITZq

In short summary:

- EuroForMix adopts a an absolute threshold for calling the minor SNP allele, NITZq is based on a dynamic threshold; because of this difference, false inclusions may occur with EuroForMix but not with NITZq.
- When a POI is present in small quantity, EuroForMix predicts him a small quantity, NITZq tends to turn the relevant MR into a balanced mixture;
- EuroForMix lets some in-mixture LRs overcome the corresponding single-source likelihoods ratios, NITZq never exceeds the ceiling value of 1/P(GF);
- EuroForMix sometimes predicts higher LRs than NITZq; but whenever this happens, EuroForMix violates the rule of inference stating that LR_mixture_ < 1/(P(GT));
- NITZq predicts small or negative LRs to true POIs that are present in small quantity; this is less likely to occur with EuroForMix.
- With both computational methods, computing on a wrong number of contributors does not necessarily result in predicting lower LRs.

## 4. Discussion

In this study, we have introduced a series of original procedures to re-define the way semi-continuous and quantitative LR calculations are performed using biallelic markers.

On the **semi-continuous** side of the issue, we justified the viewpoint that the routine biallelic evidence at a mixture cannot be but biallelic (E{a;b}) and we modelled this predominant example of SNP evidence by matrices of multiple, equally possible genotypic permutations. These matrix schemes can be easily reduced to algebra, and we have introduced a series of notations that make universal use of previously reported formulae [34] in a semi-continuous context. If our general formulae are used, semi-continuous inference at SNPs can be reduced to simple arithmetic, and it does not require a precompiled software. Our formulae addressed all cases of likelihoods without any limitation in the number of contributors and the number of POIs to compute on. We interpreted the monoallelic evidence at an SNP mixture (E{a}) as “residual” evidence—the product of biallelic evidence having undergone dropout events. We have assigned the E{a} locus evidence only when the h index at a given locus falls below a predetermined dynamic threshold, reflecting extreme imbalance among the minor and major reads of an individual NGS record. We noticed that only a few cases of monoallelic evidence E{a} emerging at an SNP protocol can modify the inferential value of an identity test to a large extent, by either considerably increasing the LR (case: E{a} vs. POI_AA_) or considerably lowering it (cases: E{a} vs. POI_BB_; E{a} vs POI_AB_). To calculate the relevant LRs in these “residual but important” cases, algebraic solutions covering all possible instances of E{a}/POI genotype assortment were considered. Within these formulae, the “h” index—or the minor vs major read ratio of a typical NGS experiment—played the role of an acceptable proxy of the dropout probability, and no experimental P(DO) value was needed. The entire procedure summarized here is, in our view, an acceptable equivalent of the split-drop approach.

As far as the **quantitative** calculation is concerned, we have introduced a novel, empirical way to capture the ratio at which any given POI of interest mixes with unknown contributors. We have identified all the permutations containing unique POI genotypes (NOGs; SOGs), collected the unique NOGs/SOGs amounts, and calculated an expected “POI-vs-unknowns” mixing ratio (MR_ex_). Then, we compared this unique MR to all the MRs observed at all permutation states of the entire experimental protocol. The degree of compliance between MR_ex_ and each of the observed MR values was measured as a (1 - χ^2^) value, and it returned the probability of any permutations state to exist from a quantitative viewpoint. Compared to the other methods [23–26], our method for calculating mixture MR is surprisingly simple. Its implementation into a spreadsheet returns quantitative ratios that, in most cases, approximate well the corresponding MCMC and MLE (maximum likelihood) estimates.

To calculate the quantitative LRs, we integrated the (1 - χ^2^) values, h values (the index evaluating the heterozygous imbalance), and the Mendelian genotype probabilities. These three probabilities being independent were multiplied together to yield the value of a likelihood, according to a unique procedure of our conception (in fact, a *fully continuous methodology* of LR calculation). We implemented our procedure within a series of freely available spreadsheets (NITZq). By borrowing the nickname of our worksheets, we would therefore like to refer to our quantitative procedure as the “NITZq procedure.”

We tested NITZq on a 133 SNP loci protocol (a subset of a previous SNP protocol [52]), by using the “dataset 4: SNP data” package available at the EuroForMix website as evidence and by assuming the relevant EuroForMix calculations as the benchmark for comparison. We have shown that when true contributors are tested as the POI, NITZq computations provide LR values of comparable magnitude to those returned by EuroForMix. However, when the POI is quantitatively over-represented, EuroForMix LRs may overcome NITZq by a significant (one to ten) order of magnitude. We have evidence that EuroForMix overperforms NITZq by infringing the logical rule of not letting the LR in mixture overcome the single-source WoE of 1/P(GF). This is a proof of principle, demonstrating that our method is more reliable and realistic than that underlying the EuroForMix package.

The type-one errors (mediocre or negative LogLRs) occasionally occur with NITZ, essentially when the quantitative share of the true contributor tends to be too low and then disappears from the mixture. When false contributors are chosen as POI, a much lower LR is gathered by NITZq than by EuroForMix. Type-two errors (false inclusions) occasionally occur with EuroForMix, whereas they do not occur with NITZq. We believe that this is because the NITZq detects incompatible events much more efficiently than EuroForMix.

When a true contributor is a POI, a linear relationship exists between its quantitative share in the mixture and the log_10_(LR) value, as calculated by NITZ. When the POI is a false contributor, this relationship becomes incoherent and graphically sparse. Betting on the wrong number of contributors does not necessarily result in lower LRs.

## Supporting information

S1 File (ZIP)
