# Supplementary material for: A novel computational strategy to predict the value of the evidence in the SNP-based forensic mixtures
Source: PLoS One. 2021 Oct 15;16(10):e0247344. doi: 10.1371/journal.pone.0247344 (PMC8519470; doi:10.1371/journal.pone.0247344)
Supplement: S1 File — (ZIP) [file pone.0247344.s001.zip › supp info files/Supplementary material.docx]

**Supplementary material:**

Formulae for quantitative likelihoods

If just one POI is assumed to exist, likelihoods are four at whatever mixture scheme. Here we report the algebraic treatment applying to two mixture schemes (2PM; 3PM).

**2PM scheme:**

**P(E{Q_a_,Q_b_}| POI=AA, n=2, Ƞ=1)**, or synthetically **P(AA+U)** = f_b_^2^ _*_ (1-𝜒^2^_I_) + 2f_a_f_b_ _*_(1-𝜒^2^_II_)

The relevant permutations are, in the order (the POI is in round brackets):

(AA) BB; (AA) AB

**P(E{Q_a_,Q_b_}| POI=BB, n=2, Ƞ=1)**, synthetically **P(BB+U)** **=**  f_a_^2^_*_ (1-𝜒^2^_Irev_)+ [(2Q_b_/3)/Q_a_] _*_ 2f_a_f_b_ _*_ (1-𝜒^2^_IVrev_)

The relevant permutations are, in the order (the POI is in round brackets):

(BB) AA; (BB) AB

**P(E{Q_a_,Q_b_}| POI=AB, n=2, Ƞ=1)**, synthetically **P(AB+U)** f_a_^2^ _*_ (1-𝜒^2^_IIrev_) + [(2Q_b_/3)/Q_a_ ]_*_ f_b_^2^ _*_ (1-𝜒^2^_IV_) + (Q_b_/Q_a_) _*_2f_a_f_b_

The relevant permutations are, in the order (the POI is in round brackets):

(AB) AA; (AB) BB; (AB) AB

**P(E{Q_a_,Q_b_}| n=2), synthetically P(U+U)** =

f_a_^2^_*_ f_b_^2^_*_ [(1-𝜒^2^_I_)+ (1-𝜒^2^_Irev_)] + 2f_a_^3^f_b_ _*_ [(1-𝜒^2^_II_)+(1-𝜒^2^_IIrev_)]+

[(2Q_b_/3)/Q_a_] _*_ 2f_a_f_b_^3^ _*_ [(1-𝜒^2^_IV_) +(1-𝜒^2^_IVrev_)]+

(Q_b_/Q_a_) _*_4f_a_^2^f_b_^2^_*_ (1-𝜒^2^_v_)

The relevant permutations are, in the order:

AA BB; BB AA; AA AB; AB AA; AB BB; BB AB; AB AB

The observed values for the (1-𝜒^2^) statistics appearing in these four likelihoods are:

AA+BB

(1-𝜒^2^_I_); MR_obs I_ = Q_a_; Q_b_

BB+AA

(1-𝜒^2^_I_rev_)= Q_b_ ;Q_a_;

AA+AB

(1-𝜒^2^_II_); MR_obs II_ = (Q_a_-Q_b_ ); (2_*_Q_b_)

AB+AA;

(1-𝜒^2^_II_rev_); (2_*_Q_b_) ; (Q_a_-Q_b_ )

AB+BB

(1-𝜒^2^_IV_); MR_obs IV_ = [Q_a_+ (Q_b_ /3)]; (2Q_b_ /3)

BB+AB;

(1-𝜒^2^_IVrev_)= (2Q_b_ /3) ; [Q_a_+ (Q_b_ /3)]

AB+AB

(1-𝜒^2^_v_); MR_obs V_ = 1

For the calculation of expected values, see chapter “Finding the ‘expected’ mixture ratio of a trace”.

The following LRs are possible: AA+U/U+U; BB+U/U+U; AB+U/U+U.

**3PM scheme:**

**P(E{Q_a_,Q_b_}| POI=AA, n=3, Ƞ=1)**, or synthetically **P(AA+U+U) =**  2_*_[2f_a_^3^f_b_ _*_ (1- 𝜒^2^_II_)] **+**  2_*_[f_a_^2^ _*_ f_b_^2^ _*_ (1-𝜒^2^_I_)]  **+** {[ Q_b_^2^/(Q_a_+Q_b_)] / [Q_a_^2^/(Q_a_+Q_b_)]} _*_ 2f_a_f_b_^3^ _*_ [(1-𝜒^2^_IIIα_) +(1-𝜒^2^_IIIβ_)] **+** 4f_a_^2^f_b_^2^ _*_ (1-𝜒^2^_II_) **+** f_b_^4^_*_ (1-𝜒^2^_I_)

The relevant permutations are, in the order (the POI is in round brackets):

(AA) AA AB; (AA) AB AA; (AA) AA BB; (AA) BB AA; (AA) AB BB; (AA) BB AB; (AA) AB AB; (AA) BB BB

**P(E{Q_a_,Q_b_}| POI=AB, n=3, Ƞ=1)**, or synthetically **P(AB+U+U) =**

f_a_^4^ _*_ (1-𝜒^2^_IIrev_) **+** 2_*_ [2f_a_^3^f_b_ _*_ (1-𝜒^2^_IIrev_)] **+** {[ Q_b_^2^/(Q_a_+Q_b_)] / [Q_a_^2^/(Q_a_+Q_b_)]} _*_ f_a_^2^ _*_ f_b_^2^ _*_ [(1-𝜒^2^_IIIγ_) **+** (1-𝜒^2^_IIIδ_)] **+** 2_*_ [(Q_b_/2)/ (Q_a_)]_*_ 2f_a_f_b_^3^ _*_ (1-𝜒^2^_IV_) **+** [(Q_b_/5)/Q_a_]_*_ f_b_^4^ _*_ (1-𝜒^2^_IV_) **+**  [(Q_b_/Q_a_)_*_ 4f_a_^2^f_b_^2^ _*_ (1-𝜒^2^_V_)]

The relevant permutations are, in the order (the POI is in round brackets):

(AB) AA AA; (AB) AA AB; (AB) AB AA; (AB) AA BB; (AB) BB AA; (AB) AB BB; (AB) BB AB; **(AB)+BB+BB;** (AB) AB AB

**P(E{Q_a_,Q_b_}| POI=BB, n=3, Ƞ=1)**, or synthetically **P(AA+U+U) =**

f_a_^4^ _*_ (1-𝜒^2^_Irev_) **+** 2_*_[ f_a_^2^ _*_ f_b_^2^ _*_ (1-𝜒^2^_Irev_)] **+** { [ Q_b_^2^/(Q_a_+Q_b_)]/ [Q_a_^2^/(Q_a_+Q_b_)] } _*_ 2f_a_^3^f_b_ _*_  [(1-𝜒^2^_IIIε_) + (1-𝜒^2^_IIIζ_)] +2_*_ [(Q_b_/5)/Q_a_]_*_ 2f_a_f_b_^3^ _*_ (1-𝜒^2^_IVrev_) **+** [(Q_b_/2)/ (Q_a_)]_*_ 4f_a_^2^f_b_^2^ _*_ (1-𝜒^2^_IVrev_)

The relevant permutations are, in the order (the POI is in round brackets):

(BB) AA AA; (BB) AA BB; (BB) BB AA; (BB) AA AB; (BB) AB AA;; (BB) AB BB; (BB) BB AB; (BB) AB AB

**P(E{Q_a_,Q_b_}| n=3)**, or synthetically **P(U+U+U)=**

2_*_ [2f_a_^5^f_b_ _*_ (1- 𝜒^2^_II_)]+ 2_*_[f_a_^4^ _*_ f_b_^2^ _*_ (1-𝜒^2^_I_)] + {[ Q_b_^2^/(Q_a_+Q_b_)] / [Q_a_^2^/(Q_a_+Q_b_)] } _*_ 2f_a_^3^ _*_ f_b_^3^ _*_ [ (1-𝜒^2^_IIIα_)+ (1-𝜒^2^_IIIβ_)+ (1-𝜒^2^_IIIγ_)+ (1-𝜒^2^_IIIδ_)+ (1-𝜒^2^_IIIε_)+ (1-𝜒^2^_IIIζ_)] + 2_*_ [4f_a_^4^ _*_ f_b_^2^ _*_ (1-𝜒^2^_IIrev_)] + 2_*_ [(Q_b_/2)/Q_a_] _*_ 4f_a_^2^ _*_ f_b_^4^ _*_ (1-𝜒^2^_IV_) + 2* [f_a_^2^ _*_ f_b_^4^ _*_ (1-𝜒^2^_Irev_)] + 2_*_[(Q_b_/5)/(Q_a_)]_*_ 2f_a*_f_b_^5^ _*_ (1-𝜒^2^_IVrev_) + 4f_a_^4^_*_f_b_^2^ _*_ (1-𝜒^2^_II_) + f_a_^2^_*_f_b_^4^_*_ (1-𝜒^2^_I_) + 2f_a_^5^_*_ f_b_ _*_ (1- 𝜒^2^_IIrev_) + [(Q_b_/Q_a_) _*_ 8f_a_^3^_*_ f_b_^3^ _*_ (1-𝜒^2^_V_)]+ [(Q_b_/5)/Q_a_] _*_ 2 f_a*_ f_b_^5^_*_ (1-𝜒^2^_IV_) + f_a_^4^ _*_ f_b_^2^_*_ (1-𝜒^2^_Irev_)+ [(Q_b_/2)/Q_a_] _*_4f_a_^2^_*_ f_b_^4^ _*_ (1-𝜒^2^_IVrev_)

The relevant permutations are, in the order:

AA AA AB; AA AB AA; AA AA BB; AA BB AA; AA AB BB; AA BB AB; AB AA BB; AB BB AA ; BB AA AB; BB AB AA; AB AA AB; AB AB AA; AB AB BB ; AB BB AB ; BB AA BB; BB BB AA; BB AB BB ; BB BB AB ; AA AB AB; AA BB BB; AB AA AA; *AB AB AB*; AB BB BB; BB AA AA; BB AB AB

The observed values for the (1-𝜒^2^) statistics appearing in these four likelihoods are:

AA+BB+AA; AA+AA+BB; AA+BB+BB

(1-𝜒^2^_I_)]; MR_obs I_ = Q_a_/(Q_a_+Q_b_); Q_b_/(Q_a_+Q_b_);

BB+AA+AA; BB+AA+BB; BB+BB+AA

(1-𝜒^2^_I_rev_); MR_obs Irev_ = Q_b_/(Q_a_+Q_b_); Q_a_/(Q_a_+Q_b_)

(AA+AA+AB;AA+AB+AA;AA+AB+AB);

(1-𝜒^2^_II_); MR_obs II_ = [(Q_a_-Q_b_)/(Q_a_+Q_b_)]; [2Q_b_/(Q_a_+Q_b_)];

AB+AB+AA;AB+AA+AB; AB+AA+AA

(1-𝜒^2^_II_rev_); MR_obs IIrev_ = [2Q_b_/(Q_a_+Q_b_)]; [(Q_a_-Q_b_)/(Q_a_+Q_b_)]

AA+AB+BB

(1-𝜒^2^_IIIα_); MR_obs IIIα_ = [(Q_a_+Q_b_) - Q_AB_ ]/2 ; {[Q_a_^2^/(Q_a_+Q_b_)] + [Q_b_^2^/(Q_a_+Q_b_)]}; [(Q_a_+Q_b_) - Q_AB_ ]/2

AA+BB+AB

(1-𝜒^2^_IIIβ_); MR_obs IIIβ_ = [(Q_a_+Q_b_) - Q_AB_]/2 ; [(Q_a_+Q_b_) - Q_AB_ ]/2; {[Q_a_^2^/(Q_a_+Q_b_)] + [Q_b_^2^/(Q_a_+Q_b_)]};

AB+AA+BB

(1-𝜒^2^_IIIγ_); MR_obs IIIγ_ = {[Q_a_^2^/(Q_a_+Q_b_)] + [Q_b_^2^/(Q_a_+Q_b_)]}; [(Q_a_+Q_b_) - Q_AB_ ]/2 ; [(Q_a_+Q_b_) - Q_AB_ ]/2

AB+BB+AA

(1-𝜒^2^_IIIδ_); MR_obs IIIδ_ = {[Q_a_^2^/(Q_a_+Q_b_)] + [Q_b_^2^/(Q_a_+Q_b_)]}; [(Q_a_+Q_b_) - Q_AB_ ]/2; [(Q_a_+Q_b_) - Q_AB_ ]/2 ;

BB+AA+AB

(1-𝜒^2^_IIIε_); MR_obs IIIε_ = [(Q_a_+Q_b_) - Q_AB_ ]/2; [(Q_a_+Q_b_) - Q_AB_ ]/2 ; {[Q_a_^2^/(Q_a_+Q_b_)] + [Q_b_^2^/(Q_a_+Q_b_)]};

BB+AB+AA

(1-𝜒^2^_IIIζ_); MR_obs IIIε_ = [(Q_a_+Q_b_) - Q_AB_ ]/2; ; {[Q_a_^2^/(Q_a_+Q_b_)] + [Q_b_^2^/(Q_a_+Q_b_)]}; [(Q_a_+Q_b_) - Q_AB_ ]/2

AB+BB+BB; AB+AB+BB;AB+BB+AB

(1-𝜒^2^_IV_); MR_obs IV_ = [Q_a_+(Q_b_/5)]; +[(4_*_Q_b_)/5];

BB+AB+AB; BB+AB+BB; BB+BB+AB

(1-𝜒^2^_IVrev_); MR_obs IVrev_ = [(4_*_Q_b_)/5]; [Q_a_+(Q_b_/5)];

AB+AB+AB

(1-𝜒^2^_v_); MR_obs V_ =1

For the calculation of expected values, see chapter “Finding the ‘expected’ mixture ratio of a trace”.

The following LRs become possible: AA+U+U/U+U+U; BB+U+U/U+U+U; AB+U+U/U+U+U.

Likelihood calculations involving one POI and four contributors have been implemented into three worksheets to be downloaded from [www.vincepascali.it/docs](http://www.vincepascali.it/docs)

Flow chart of NITZq calculations

NITZq worksheets flowchart 133 SNP loci and they calculate semicontinuous and quantitative LRs.

To use NITZq the following steps are recommended.

**I. evidence input.** The appropriate worksheet of the NITZq files (NITZq 2PM, NITZq 3PM and NITZq 4PM) should be selected. Each worksheet has an “*input & MR*” spreadsheet that prompts the user to input the following data (from the worksheet’s top-left side onwards and in sequential order per each locus): locus name; major read; minor read; gene frequency of the major read gene; gene frequency of minor read gene;

**II. POI genotypes input**; all POI(s) genotypes have first to be code-classified as AA, BB, AB according to the conventional, major-read allele / minor-read allele code. Encoded genotypes are then paste into the input area as ‘reference data’ in the area placed adjacent to the ‘evidence data’. This will trigger calculations into a series of cross-referenced worksheets.

**III. basic calculations:** specific algebraic functions placed in a “*calculations”* worksheet calculate genotype frequencies, individual quantities and heterozygous ratio (h) at every record corresponding to each permutation state of the matrices.

**IV. calculation of the MR:** at the “input & MR” spreadsheet, NOGS/SOGs values are intercepted based on the POI genotypes. Their values are summed and averaged and the quantity fraction belonging to the POI is deducted. Finally, (1-Q_POI_) divided by the number of unknowns will give the other fractions of MR. The array of the MR values is cross-referenced into the appropriate positions at the ‘*calculations’* worksheet.

**V. (1- χ^2^) calculation:** the individual quantities deducted at each permutation state are compared to the NOGs/SOGs MR_exp_ and transformed into (1- χ^2^) values (one value per permutation);

**VI likelihood calculations**: genotype frequencies, h values and (1- χ^2^) values are multiplied across each permutation state and their products summed together to give the likelihood of choice.

**VII calculation of the LR**

Likelihood and LR calculations update instantly as soon as the evidence and POI input is pasted into the “input & MR” spreadsheet.

**VIII correction for incompatible cases**

Extremely imbalanced read pairs in the evidence may imply that all mixture contributors are homozygous and that the evidence itself is single-allele (E{a}). These cases need to be identified and brought back to algebraic calculations. To identify these cases we calculate the h index at every locus evidence, set a threshold for the h-evidence value below which the locus is declared ‘single-allele’ and then we reclassify the evidence as single-allele. Identifying single-allele evidence is essential in order to obtain the appropriate LR value.
